# Circulating virome and inflammatory proteome in patients with ST-elevation myocardial infarction and primary ventricular fibrillation

**DOI:** 10.1038/s41598-022-12075-x

**Published:** 2022-05-12

**Authors:** Teresa Oliveras, Elena Revuelta-López, Cosme García-García, Adriana Cserkóová, Ferran Rueda, Carlos Labata, Marc Ferrer, Santiago Montero, Nabil El-Ouaddi, Maria José Martínez, Santiago Roura, Carolina Gálvez-Montón, Antoni Bayes-Genis

**Affiliations:** 1 Heart Failure Unit and Cardiology Department, Hospital Universitari Germans Trias i Pujol, Carretera de Canyet s/n, Badalona, 08916 Barcelona, Spain; 2 Heart Failure and Cardiac Regeneration (ICREC) Research Program, Health Sciences Research Institute Germans Trias i Pujol (IGTP), Badalona, Barcelona, Spain; 3 CIBERCV, Instituto de Salud Carlos III, Madrid, Spain; 4 Faculty of Medicine, University of Vic-Central University of Catalonia (UVic-UCC), Vic, Barcelona, Spain; 5 Department of Medicine, Universitat Autònoma de Barcelona, Barcelona, Spain; 6 Heart Institute, Hospital Universitari Germans Trias i Pujol, Carretera de Canyet s/n, Badalona, 08916 Barcelona, Spain

**Keywords:** Cardiology, Medical research

## Abstract

Primary ventricular fibrillation (PVF) is a life-threatening complication of ST-segment elevation myocardial infarction (STEMI). It is unclear what roles viral infection and/or systemic inflammation may play as underlying triggers of PVF, as a second hit in the context of acute ischaemia. Here we aimed to evaluate whether the circulating virome and inflammatory proteome were associated with PVF development in patients with STEMI. Blood samples were obtained from non-PVF and PVF STEMI patients at the time of primary PCI, and from non-STEMI healthy controls. The virome profile was analysed using VirCapSeq-VERT (Virome Capture Sequencing Platform for Vertebrate Viruses), a sequencing platform targeting viral taxa of 342,438 representative sequences, spanning all virus sequence records. The inflammatory proteome was explored with the Olink inflammation panel, using the Proximity Extension Assay technology. After analysing all viral taxa known to infect vertebrates, including humans, we found that non-PVF and PVF patients only significantly differed in the frequencies of viruses in the *Gamma-herpesvirinae* and *Anelloviridae* families. In particular, most showed a significantly higher relative frequency in non-PVF STEMI controls. Analysis of systemic inflammation revealed no significant differences between the inflammatory profiles of non-PVF and PVF STEMI patients. Inflammatory proteins associated with cell adhesion, chemotaxis, cellular response to cytokine stimulus, and cell activation proteins involved in immune response (IL6, IL8 CXCL-11, CCL-11, MCP3, MCP4, and ENRAGE) were significantly higher in STEMI patients than non-STEMI controls. CDCP1 and IL18-R1 were significantly higher in PVF patients compared to healthy subjects, but not compared to non-PVF patients. The circulating virome and systemic inflammation were not associated with increased risk of PVF development in acute STEMI. Accordingly, novel strategies are needed to elucidate putative triggers of PVF in the setting of acute ischaemia, in order to reduce STEMI-driven sudden death burden.

## Introduction

In cases of acute myocardial infarction with ST-segment elevation (STEMI), morbidity and mortality have substantially decreased following the establishment of regional and national reperfusion networks, and the use of newer evidence-based drugs^1,2^. However, ventricular fibrillation (VF) during the acute phase of myocardial infarction—also known as primary ventricular fibrillation (PVF)—is still the leading cause of sudden prehospital cardiac death, and is a factor that predicts poor short-term prognosis^3,4^. In this context, numerous studies have attempted to identify predictors of PVF, without significant progresses. It is likely that susceptibility to VF during acute ischemia might be modulated by several factors, including hemodynamic dysfunction, electrolyte alterations, autonomic dysregulation, genetic factors, and certain environmental influences.

Particularly, an association between viral infections and acute myocardial infarction (AMI) has been proposed^5,6^. Furthermore other authors have found seasonal variations in sudden cardiac death (SCD)^7^, typically with a peak in winter, suggesting that viral exposure is a trigger of VF in patients suffering from acute ischemia^8^. However, only influenza virus and some enteroviruses have been investigated for roles in SCD, with contradictory results^9,10^. To date, evidence is also scarce and unclear regarding an association between inflammatory biomarkers and SCD in an asymptomatic population. For instance, interleukin 6 (IL-6) is reported as a predictor of sudden death in healthy men^11^. Additionally, growth differentiation factor 15 (GDF-15) has been described as a risk factor for SCD during the acute phase of myocardial infarction^12^, and as a predictor of short-term mortality in patients with PVF^13^. Nevertheless, the association between systemic inflammation at the time of STEMI and PVF remains unknown.

In the present study, we aimed to conduct a pilot study with two objectives: (1) to examine the circulating virome sequence, and (2) to explore the systemic inflammatory proteome in patients with STEMI, with and without PVF.

## Methods

### Patient population

The RUTI-STEMI-PVF cohort is a prospective single-centre registry of consecutive STEMI patients treated with primary percutaneous coronary intervention (PCI) and within the Codi IAM reperfusion network^14,15^. STEMI was defined according to the Third Universal Definition of Myocardial Infarction^16^. Patient management was decided by the physicians, following recommended guidelines^17,18^. Upon admission, blood samples were obtained by venipuncture and centrifuged, and then heparin-plasma was stored at − 80 °C until assay.

Patients were divided into two groups: those who had suffered PVF, and those who had not (non-PVF). PVF was defined as ventricular fibrillation occurring ≤ 24 h after diagnosis of myocardial infarction, and not preceded by heart failure or shock.

Blood samples for virome analyses were obtained from non-PVF (*n* = 9) and PVF (*n* = 11) STEMI patients at the time of primary PCI. The patients had a mean age of 60 ± 10 years and were 85% men. Patients with a first STEMI were selected, matched by sex, age, diabetes, and anterior myocardial infarction. Table 1 shows clinical and demographic characteristics of the studied groups. The inflammatory proteome was analysed among the same patients with available samples, PVF and non-PVF, as well as in non-STEMI healthy controls (without history of cardiovascular disease or cancer; mean age, 58.6 ± 1.2 years, 60% men). Written informed consent was obtained from all patients. The study was approved by the local ethics committee (The Ethics Committee of the Clinical Investigation of Germans Trias i Pujol Hospital) and was conducted in accordance with the Declaration of Helsinki.

**Table 1:** Clinical characteristics of PVF and non-PVF STEMI patients.

Variable	PVF STEMI (n = 11)	non-PVF STEMI (n = 9)	*P* value
Age, years, mean (standard deviation)	59.5 (11.7)	60.2 (9)	0.889
Male sex, n (%)	9 (81.8%)	8 (88.9%)	0.579
**Medical history, n (%)**			
Hypertension	5 (45.5%)	6 (66.7%)	0.311
Hyperlipidaemia	6 (54.5%)	7 (77.8%)	0.272
Diabetes mellitus	2 (18.2%)	2 (22.2%)	0.625
Current smoker	7 (63.6%)	6 (66.7%)	0.630
Persistent or permanent atrial fibrillation	1 (9.1%)	0	0.550
**Previous treatment, n (%)**			
Aspirin	0	1 (11.1%)	0.450
Beta-blocker	0	1 (11.1%)	0.450
Statin	2 (18.2%)	1 (11.1%)	0.579
Angiotensin-converting enzyme inhibitor or angiotensin II receptor blocker	1 (9.1%)	2 (22.2%)	0.421
**Clinical characteristics**			
At least 2 angina episodes in the last 24 h, n (%)	3 (27.3%)	1 (11.2%)	0.375
Killip–Kimball class, n (%)			
I	6 (54.4%)	8 (88.9%)	
II	1 (9.1%)	1 (11.1%)	
III	0	0	
IV	4 (36.4%)	0	
Killip–Kimball > 1	5 (45.5%)	1 (11.1%)	0.119
**ECG characteristics**			
Anterior STEMI, n (%)	7 (63.6%)	4 (44.4%)	0.342
Atrial fibrillation on first ECG, n (%)	3 (27.3%)	0	0.145
**Echocardiography**			
LVEF after PCI, %, median (IQR)	42 (40–55)	56 (52–61)	0.003
**Culprit lesion, n (%)**			
Left anterior descending artery	5 (45.5%)	3 (33.3%)	0.465
Circumflex artery	0	2 (22.2%)	0.189
Right coronary artery	5 (45.5%)	4 (44.4%)	0.658
Left main coronary artery	1 (9.1%)	0	0.550
Multivessel disease, n (%)	7 (63.6%)	3 (33.3%)	0.185
Primary percutaneous coronary intervention, n (%)	10 (90.9%)	9 (100%)	0.550
Complete revascularization, n (%)	5 (45.5%)	5 (55.6%)	0.500
**Timing of procedure**			
Symptoms onset to first medical contact, minutes, median (IQR)	18 (10–25)	45 (27–170)	0.053
Symptoms onset to PPCI, minutes, median (IQR)	135 (107–208)	144 (113–261)	0.543
Symptoms onset to PPCI < 120 min, n (%)	7 (63.6%)	7 (77.8%)	0.426
**Clinical events during hospitalization, n (%)**			
Recurrent ischemic event	1 (9.1%)	0	0.550
Atrial fibrillation or flutter	2 (18.2%)	1 (11.1%)	0.579
Sustained ventricular tachycardia	1 (9.1%)	0	0.550
Post-anoxic encephalopathy	7 (63.6%)	0	0.004
Intrahospital mortality	5 (45.5%)	0	0.030

### Nucleic acid extraction

DNA was purified from total blood collected in BD Vacutainer EDTA tubes, using the FlexiGene DNA Kit (QIAGEN GmbH, Germany). DNA concentration was evaluated using the Qubit BR Assay Kit (Thermo Scientific, Wilmington, DE, USA). Integrity was checked by gel electrophoresis.

### Molecular assays

We analysed a total of 20 human blood samples, from 9 patients with STEMI and non-PVF, and from 11 patients with STEMI and PVF, using VirCapSeq-VERT (Virome Capture Sequencing Platform for Vertebrate Viruses). VirCapSeq-VERT is a virome capture sequencing platform targeting viral taxa that infect vertebrates, using a database of 342,438 representative sequences spanning all virus sequence records^19^. When compared with other enrichment procedures, the utilized procedure allows for reduction of background human DNA, as well as a 100- to 10,000-fold enrichment in viral reads. This system enables the identification and genetic characterization of all known vertebrate viruses and their genetic variants (the genomes of 207 viral taxa known to infect vertebrates, including humans). Samples were processed using Illumina HiSeq/NovaSeq.

### Bioinformatics data analysis

Bioinformatic data analysis involved the following workflow: identify and remove host background reads, quality check and trimming, de novo assembly, homology search for putative viral genomes, mapping of filtered reads and generation of counts, and analysis of viral communities. Kraken tools were used to remove sequenced human and bacterial reads from among the total sequencing reads generated for each sample^20^. A quality check and adapter trimming were performed using the quality control tool FASTQC^21^. Assembly of the host depleted trimmed reads was performed using SPAdes software^22^ version 3.15.2. Generation of the index and the mapping was done using BWA software^23^ version 0.7.17. Amplification duplicates that might confound the count were remove using SAMtools software^24^ version 1.12. Finally, mapping statistics were generated using the MultiQC tool^25^.

### Epstein–Barr real time PCR and immunoassay

Real time PCR was performed in non-PVF (n = 6) and PVF (n = 9) DNA samples by EBV Amplification Reagent Kit (Abbott Molecular, 08N54-085).

IgG-class antibodies to Epstein–Barr virus nuclear antigen (EBV-EBNA-1) were determined in plasma. Non-PVF (n = 94) and PVF (n = 82) plasma samples were by Epstein–Barr Virus (EBNA-1) IgG ELISA (Demeditec, DE4246, lot 109G/K041).

### Inflammation proteomic analysis

The inflammatory proteomic profiles of non-PVF and PVF patients were analysed using the Olink Inflammation panel, based on Proximity Extension Assay technology. This multiplex immunoassay enables analysis of 92 inflammation-related proteins^26,27^. Non-PVF patients (*n* = 7), PVF patients (*n* = 7), and healthy subjects (*n* = 5) were analysed using the Olink Inflammation panel.

### Statistical analysis

Summary data were represented by mean and standard error of the mean (SEM), or by median and interquartile range (IQR) depending on the data normality. The D’Agostino and Pearson test was used to evaluate the normality of data. Two-groups comparisons were performed using the unpaired t-test or Mann Whitney test, and three-groups comparisons were performed using Kruskal–Wallis test or ANOVA, depending on the data normality. Fisher's exact test was used when required. Statistical significance was assumed when *P* was < 0.05. Statistical analyses were performed using Prism 9 for macOS version 9.0.2 (134) and 9.3.1 (350).

## Results

### Circulating virome

The virome capture sequencing platform VirCapSeq-VERT was used to target viral taxa in human blood samples from non-PVF patients (*n* = 9) and PVF patients (*n* = 11). The capture results were sequenced using Illumina HiSeq/NovaSeq. Human and bacterial reads were removed from the sequencing files, and the remaining reads ranged from 148–543 k pairs of reads per sample (Supplementary Table 1). Along all reads and samples, we found good quality per base position. However, we detected a high amount of PCR duplicates, due to the amplification and enrichment protocol (Supplementary Table 2). We also identified and removed common sequencing adapters. Details in the statistics regarding the trimming process for each sample are shown in Supplementary Table 3. The host depleted trimmed reads were assembled using SPAdes software to generate longer sequences, and for an additional and improved homology search (Supplementary Table 4). All viral reference genomes available in GenBank NCBI database were used to create a BLAST database, which was used for the homology search, with the generated assembled host depleted trimmed reads as input. We observed a total of 51 different genome entries (Table 2).

**Table 2:** Taxonomic information of the sequence identified.

NucGenbank	Name	RefSeq	taxID	Family	Genus	Species
NC_043061.1	Equid gammaherpesvirus 7	GCF_002814995.1	291612	Herpesviridae	Gammaherpesvirinae_unclassified	Equid gammaherpesvirus 7
NC_007605.1	Human gammaherpesvirus 4	GCF_002402265.1	10376	Herpesviridae	Lymphocryptovirus	Human gammaherpesvirus 4
NC_009334.1	Human gammaherpesvirus 4	GCF_000872045.1	12509	Herpesviridae	Lymphocryptovirus	Human gammaherpesvirus 4
NC_006146.1	Macacine gammaherpesvirus 4	GCF_000846585.1	45455	Herpesviridae	Lymphocryptovirus	Macacine gammaherpesvirus 4
NC_038859.1	Panine gammaherpesvirus 1	GCF_002985915.1	159602	Herpesviridae	Lymphocryptovirus	Panine gammaherpesvirus 1
NC_043058.1	Papiine gammaherpesvirus 1	GCF_002814855.1	106332	Herpesviridae	Lymphocryptovirus	Papiine gammaherpesvirus 1
NC_038860.1	Pongine gammaherpesvirus 2	GCF_002985945.1	159603	Herpesviridae	Lymphocryptovirus	Pongine gammaherpesvirus 2
NC_015049.1	Cricetid gammaherpesvirus 2	GCF_000892215.1	1605972	Herpesviridae	Rhadinovirus	Cricetid gammaherpesvirus 2
NC_009333.1	Human gammaherpesvirus 8	GCF_000838265.1	37296	Herpesviridae	Rhadinovirus	Human gammaherpesvirus 8
NC_001716.2	Human betaherpesvirus 7	GCF_000848125.1	10372	Herpesviridae	Roseolovirus	Human betaherpesvirus 7
NC_007822.1	Escherichia virus WA45	GCF_002618845.1	338105	Microviridae	Alphatrevirus	Escherichia virus WA45
NC_007856.1	Escherichia virus G4	GCF_000867085.1	489829	Microviridae	Gequatrovirus	Escherichia virus G4
NC_007825.1	Escherichia virus ID52	GCF_002614425.1	338108	Microviridae	Gequatrovirus	Escherichia virus ID52 Escherichia phage ID52
NC_007817.1	Escherichia virus Talmos	GCF_000864545.1	511969	Microviridae	Gequatrovirus	Escherichia virus Talmos Escherichia phageID2 Moscow/ID/2001
NC_001420.2	Coliphage	GCF_000840785.1	10843	Microviridae	Gequatrovirus	Escherichia virus G4
NC_001422.1	Escherichia virus phiX174	GCF_000819615.1	10847	Microviridae	Sinsheimervirus	Escherichia virus phiX174
NC_022518.1	Human endogenous retrovirus K113	GCF_000913595.1	166122	Retroviridae	Human endogenous retroviruses	Human endogenous retrovirus K
NC_032111.1	BeAn 58058 virus	GCF_001907825.1	67082	Poxviridae	Chordopoxvirinae_unclassified	BeAn 58058 virus
NC_008168.1	Choristoneura fumiferana granulovirus	GCF_000869805.1	56947	Baculoviridae	Betabaculovirus	Choristoneura fumiferana granulovirus
NC_026663.1	Simian Torque teno virus 30	GCF_000959655.1	1619218	Anelloviridae	Alphatorquevirus	Simian Torque teno virus 30
NC_026662.1	Simian Torque teno virus 31	GCF_000954935.1	1619219	Anelloviridae	Alphatorquevirus	Simian Torque teno virus 31
NC_026664.1	Simian Torque teno virus 32	GCF_000959695.1	1619220	Anelloviridae	Alphatorquevirus	Simian Torque teno virus 32
NC_026764.1	Simian Torque teno virus 33	GCF_000969135.1	1629656	Anelloviridae	Alphatorquevirus	Simian Torque teno virus 33
NC_026765.1	Simian Torque teno virus 34	GCF_000969075.1	1629657	Anelloviridae	Alphatorquevirus	Simian torque teno virus 34
NC_015783.1	Torque teno virus	GCF_000893775.1	68887	Anelloviridae	Alphatorquevirus_unclassified	Torque teno virus
NC_002076.2	Torque teno virus 1	GCF_000857545.1	687340	Anelloviridae	Alphatorquevirus	Torque teno virus 1
NC_014076.1	Torque teno virus 10	GCF_000887255.1	687349	Anelloviridae	Alphatorquevirus	Torque teno virus 10
NC_038338.1	Torque teno virus 11	GCF_002818275.1	687350	Anelloviridae	Alphatorquevirus	Torque teno virus 11
NC_014075.1	Torque teno virus 12	GCF_000889775.1	687351	Anelloviridae	Alphatorquevirus	Torque teno virus 12
NC_038339.1	Torque teno virus 13	GCF_002818305.1	687352	Anelloviridae	Alphatorquevirus	Torque teno virus 13
NC_014096.1	Torque teno virus 15	GCF_000889875.1	687354	Anelloviridae	Alphatorquevirus	Torque teno virus 15
NC_014091.1	Torque teno virus 16	GCF_000889855.1	687355	Anelloviridae	Alphatorquevirus	Torque teno virus 16
NC_043413.1	Torque teno virus 17	GCF_002986165.1	687356	Anelloviridae	Alphatorquevirus	Torque teno virus 17
NC_014078.1	Torque teno virus 19	GCF_000888235.1	687358	Anelloviridae	Alphatorquevirus	Torque teno virus 19
NC_038340.1	Torque teno virus 20	GCF_002818335.1	687359	Anelloviridae	Alphatorquevirus	Torque teno virus 20
NC_038341.1	Torque teno virus 21	GCF_002818355.1	687360	Anelloviridae	Alphatorquevirus	Torque teno virus 21
NC_043415.1	Torque teno virus 22	GCF_002986205.1	687361	Anelloviridae	Alphatorquevirus	Torque teno virus 22
NC_038342.1	Torque teno virus 23	GCF_002818385.1	687362	Anelloviridae	Alphatorquevirus	Torque teno virus 23
NC_038343.1	Torque teno virus 24	GCF_002818405.1	687363	Anelloviridae	Alphatorquevirus	Torque teno virus 24
NC_014079.1	Torque teno virus 26	GCF_000889795.1	687365	Anelloviridae	Alphatorquevirus	Torque teno virus 26
NC_014074.1	Torque teno virus 27	GCF_000888215.1	687366	Anelloviridae	Alphatorquevirus	Torque teno virus 27
NC_014073.1	Torque teno virus 28	GCF_000888895.1	687367	Anelloviridae	Alphatorquevirus	Torque teno virus 28
NC_038344.1	Torque teno virus 29	GCF_002818425.1	687368	Anelloviridae	Alphatorquevirus	Torque teno virus 29
NC_014081.1	Torque teno virus 3	GCF_000888935.1	687342	Anelloviridae	Alphatorquevirus	Torque teno virus 3
NC_014069.1	Torque teno virus 4	GCF_000886355.1	687343	Anelloviridae	Alphatorquevirus	Torque teno virus 4
NC_038336.1	Torque teno virus 5	GCF_002818195.1	687344	Anelloviridae	Alphatorquevirus	Torque teno virus 5
NC_014094.1	Torque teno virus 6	GCF_000888995.1	687345	Anelloviridae	Alphatorquevirus	Torque teno virus 6
NC_014080.1	Torque teno virus 7	GCF_000887275.1	687346	Anelloviridae	Alphatorquevirus	Torque teno virus 7
NC_014084.1	Torque teno virus 8	GCF_000887295.1	687347	Anelloviridae	Alphatorquevirus	Torque teno virus 8
NC_038337.1	Torque teno virus 9	GCF_002818245.1	687348	Anelloviridae	Alphatorquevirus	Torque teno virus 9
NC_043414.1	Torque tenovirus 18	GCF_002986195.1	687357	Anelloviridae	Alphatorquevirus	Torque teno virus 18

The identified genome entries were then used to create an alignment index, to map the reads corresponding to their exact position in the reference genomes. Nearly half of the reads did not map to retrieved viral sequences, likely because the host depleted reads may have contained archaea, yeast, or unclassified taxon reads. These results correspond with the low number of reads identified as viral (Supplementary Table 1). Supplementary Tables 5 and 6 summarize the length of each reference sequence, and the percent of base pairs covered (%), in non-PVF and PVF patients. Six genome entries found in the homology search did not generate reads mapping, since the better sensitivity of the mapping enabled more confident placing of a read compared to with BLAST.

### Alpha diversity

We further assessed alpha diversity to determine the diversity and to enable comparisons of the type and quantity of virus species between non-PVF and PVF patients. Alpha diversity is a statistic used in this kind of sample, in which reads reflect the abundance of each of the identified operational taxonomical units (OTUs). Richness and diversity are alpha diversity metrics.

**Figure 1:**
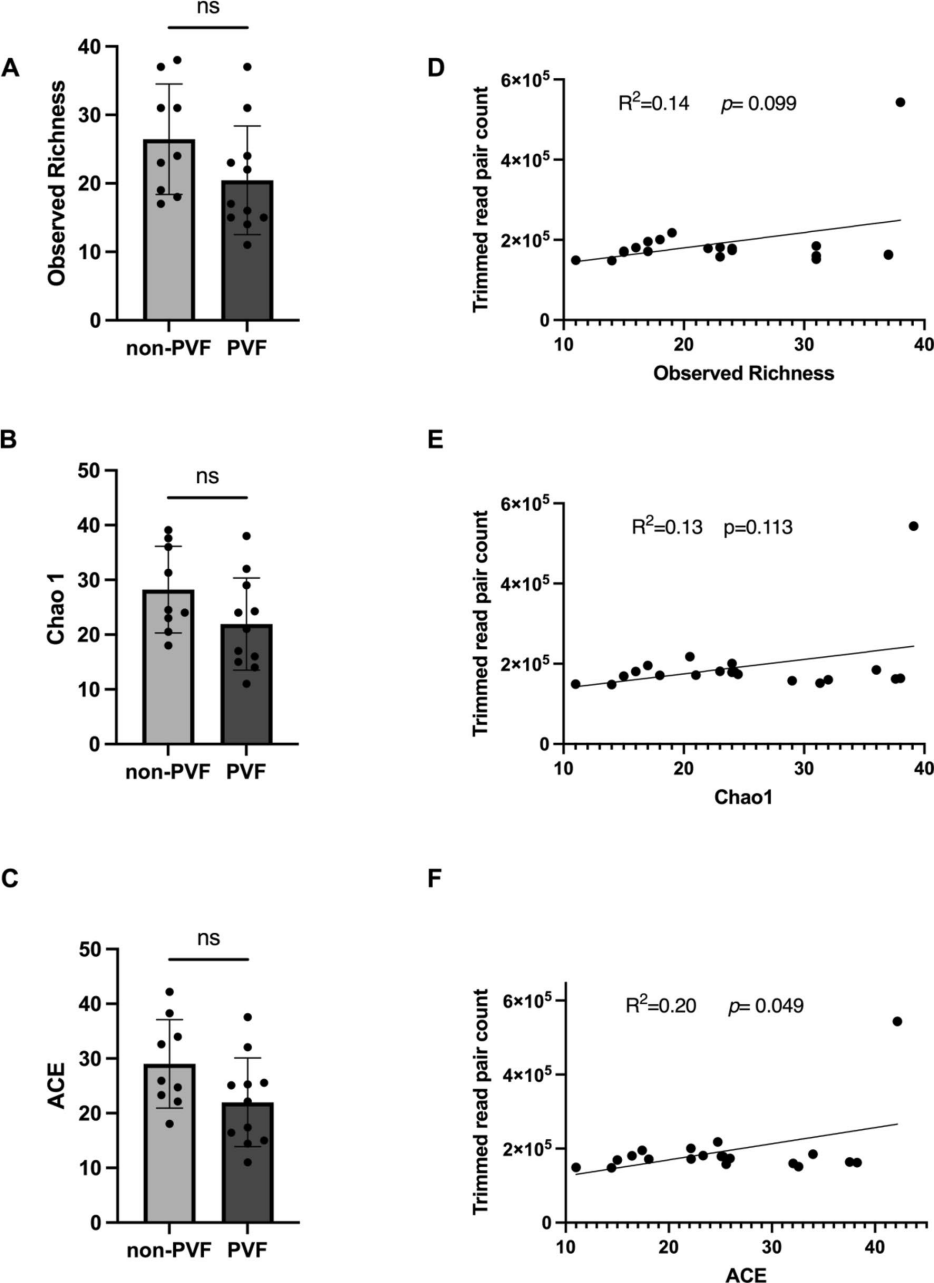
Species richness alpha diversity. (**A**–**C**) Species richness represented by the following metrics: (**A**) Observed richness values, (**B**) Chao1 Richness Estimate (Chao1), and (**C**) Abundance Coverage Estimator (ACE). (**D**–**F**) Simple linear regression model between the number of sequenced raw read pairs and (**D**) observed richness, (**E**) Chao 1, and (**F**) ACE.

**Figure 2:**
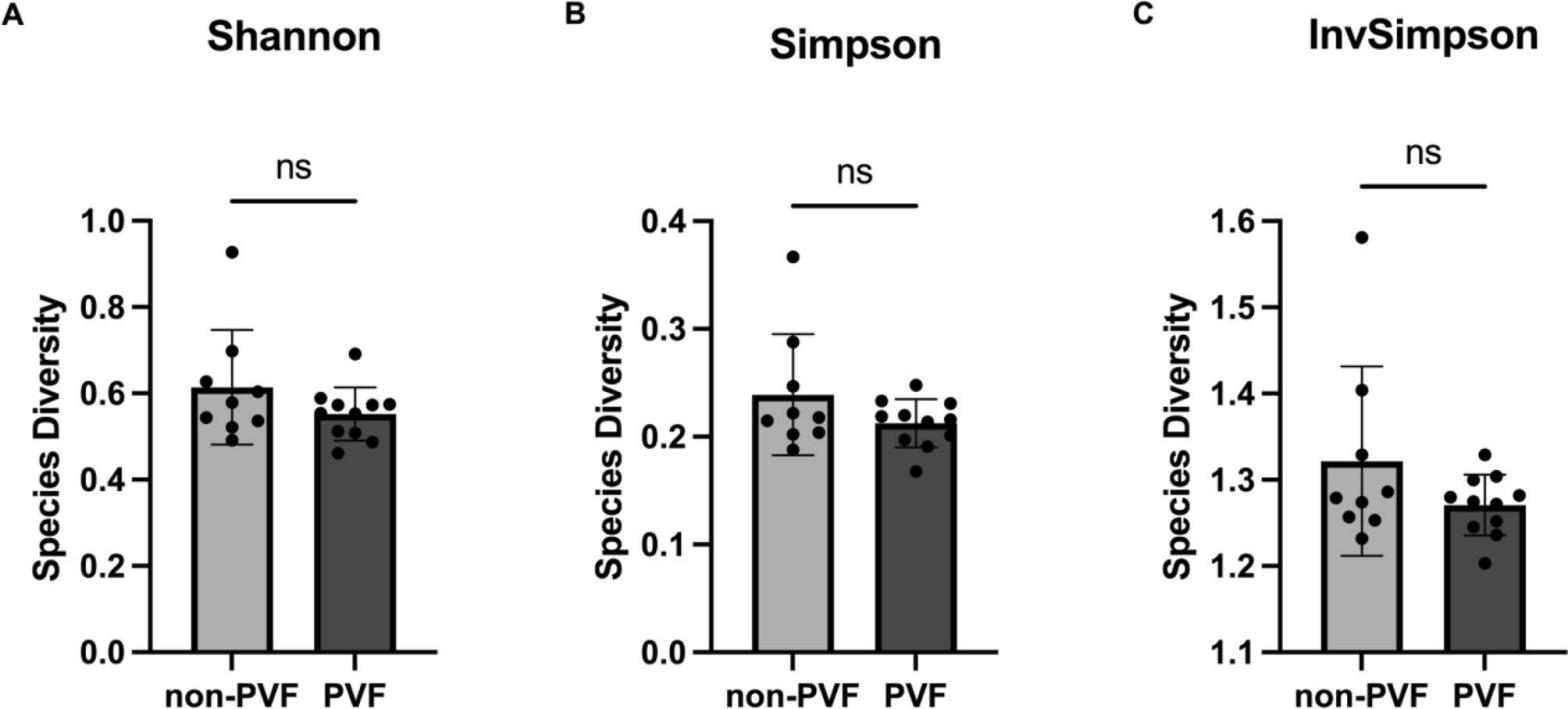
Species diversity alpha diversity. Species diversity represented by (**A**) Shannon’s Diversity Index, (**B**) the Simpson Index, and (**C**) the Inverse Simpson Index.

As a result, species richness did not significantly differ between non-PVF patients and PVF patients (26.44 ± 2.69 vs*.* 20.45 ± 2.39; *P* = 0.112) (Fig. 1A). PVF and non-PVF patients also did not significantly differ in other richness indexes, such as the Chao1 Richness Estimate (28.23 ± 2.63 vs. 21.93 ± 2.54; *P* = 0.105) (Fig. 1B) and Abundance Coverage Estimator (ACE) index (29.02 ± 2.69 vs. 22.00 ± 2.44; *P* = 0.069) (Fig. 1C). We also used a simple linear regression model to explore whether the species richness correlated with the number of raw read pairs sequenced. We identified a slight correlation between the number of raw read pairs sequenced and the observed richness (R^2^ = 0.14; *P* = 0.099) (Fig. 1D), Chao1 (R^2^ = 0.13; *P* = 0.113) (Fig. 1E), and ACE index (R^2^ = 0.20; *P* = 0.049) (Fig. 1F). Concerning the species diversity, we found no significant differences between non-PVF and PVF patients using Shannon’s Diversity Index (0.614 ± 0.044 vs. 0.552 ± 0.018; *P* = 0.252) (Fig. 2A), the Simpson Index (0.239 ± 0.019 vs. 0.212 ± 0.006; *P* = 0.456) (Fig. 2B), or the Inverse Simpson Index (1.322 ± 0.036 vs. 1.271 ± 0.011; *P* = 0.423) (Fig. 2C).

### Frequent sequences

**Figure 3:**
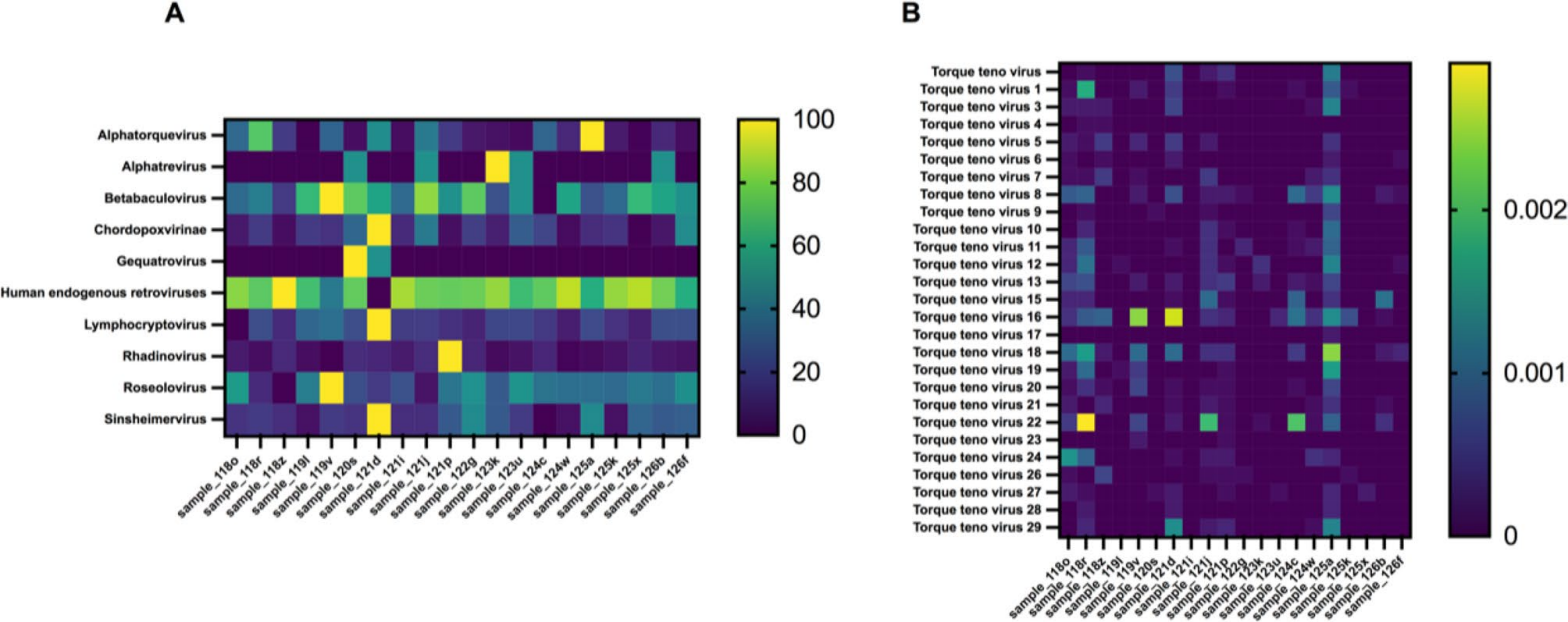
Heatmap representations of (**A**) the most frequent OTUs at genus level normalized by *Human endogenous retrovirus K113* (NC_022518.1) frequency and (**B**) the frequencies of the 27 *Torque teno virus* identified. Prism 9 for macOS version 9.3.1 (350).

In addition, due to species richness of viruses did not significantly differ between non-PVF and PVF patients, we explored whether specific virus families were differentially expressed. Supplementary Table 7 summarizes the top 10 OTUs. The predominant OTU was *Human endogenous retrovirus K113* (NC_022518.1) (Fig. 3A), and its frequency did not significantly differ between non-PVF and PVF patients (0.869 ± 0.012 vs. 0.885 ± 0.004; *P* = 0.381).

Among the most frequent OTUs, the genus most commonly found was *Lymphocryptovirus* (Fig. 3A), belonging to the *Herpesviridae* family. In particular, we detected the complete genomes of three viruses of this family—NC_007605.1, NC_009334.1, and NC006146.1—corresponding to *Human gammaherpesvirus 4* (Epstein–Barr virus), *Human herpesvirus 4 type 2* (Epstein–Barr virus type 2), and *Macacine gammaherpesvirus 4* (Rhesus lymphocryptovirus), respectively (Table 2).

**Figure 4:**
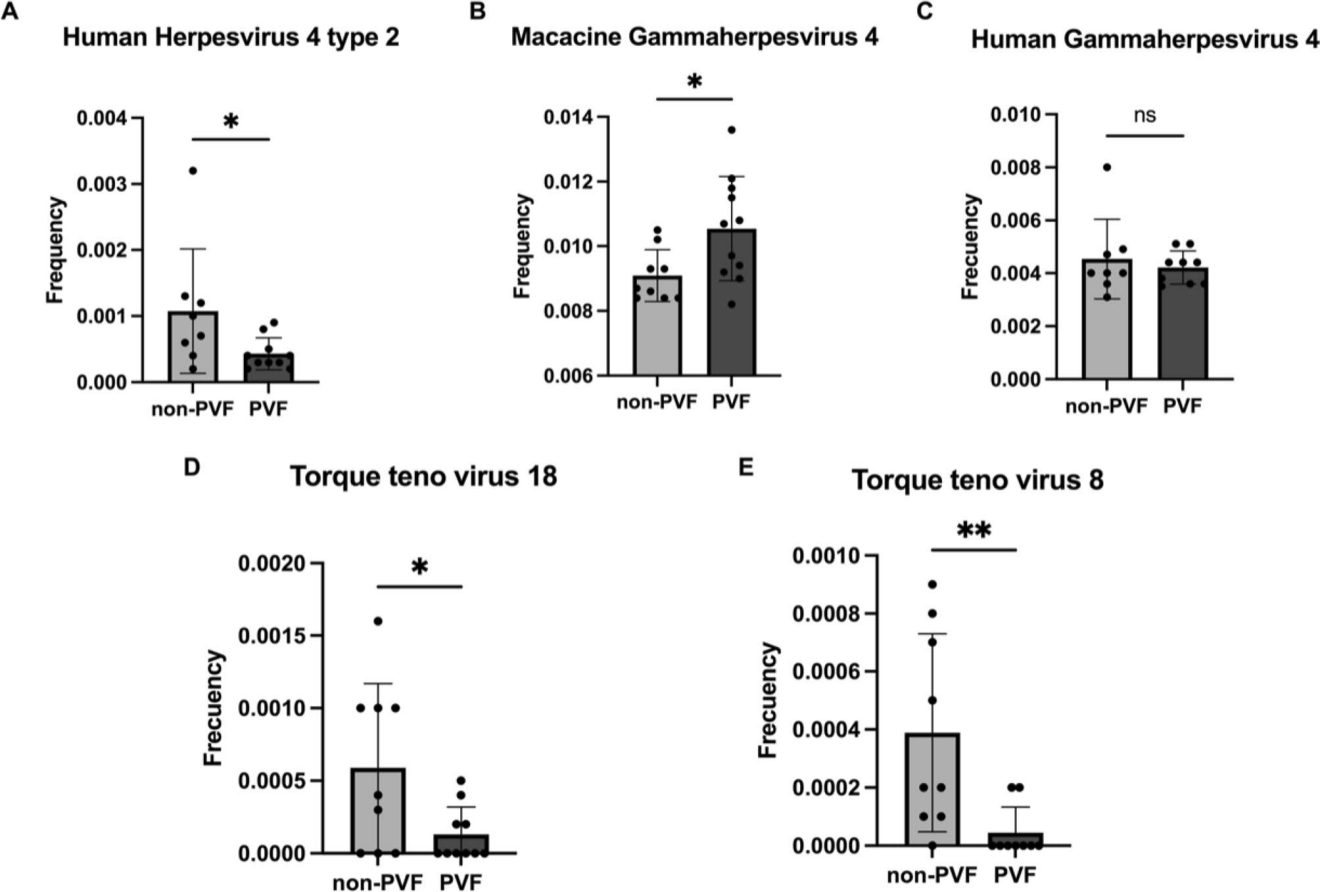
Relative frequency of (**A**) *Human herpesvirus 4 type 2* (NC_009334.1), (**B**) *Macacine gammaherpesvirus 4* (NC006146.1), (**C**) *Human gammaherpesvirus 4* (NC_007605.1), (**D**) *Torque teno virus 18* and (NC_043414.1), and (**E**) *Torque Teno virus 8* (NC_014084.1).

Non-PVF and PVF patients only significantly differed in the frequencies of *Human herpesvirus 4 type 2* and *Macacine gammaherpesvirus 4.* As shown in Fig. 4A,* Human herpesvirus 4 type 2* (NC_009334.1) was significantly more common in non-PVF patients than in PVF patients (0.0011 ± 0.000 vs. 0.0004 ± 0.000; *P* = 0.042), while *Macacine gammaherpesvirus 4* (NC_006146.1) was significantly less frequent in non-PVF patients than in PVF patients (0.009 ± 0.000 vs. 0.011 ± 0.000; *P* = 0.024) (Fig. 4B). Non-PVF and PVF patients did not significantly differ in the frequency of *Human gammaherpesvirus 4* (NC_007605.1) (0.0045 ± 0.000 vs. 0.0042 ± 0.000; *P* = 0.939) (Fig. 4C). *Human gammaherpesvirus 4* (NC_007605.1) results were also validated by RT-PCR and ELISA.

**Figure 5:**
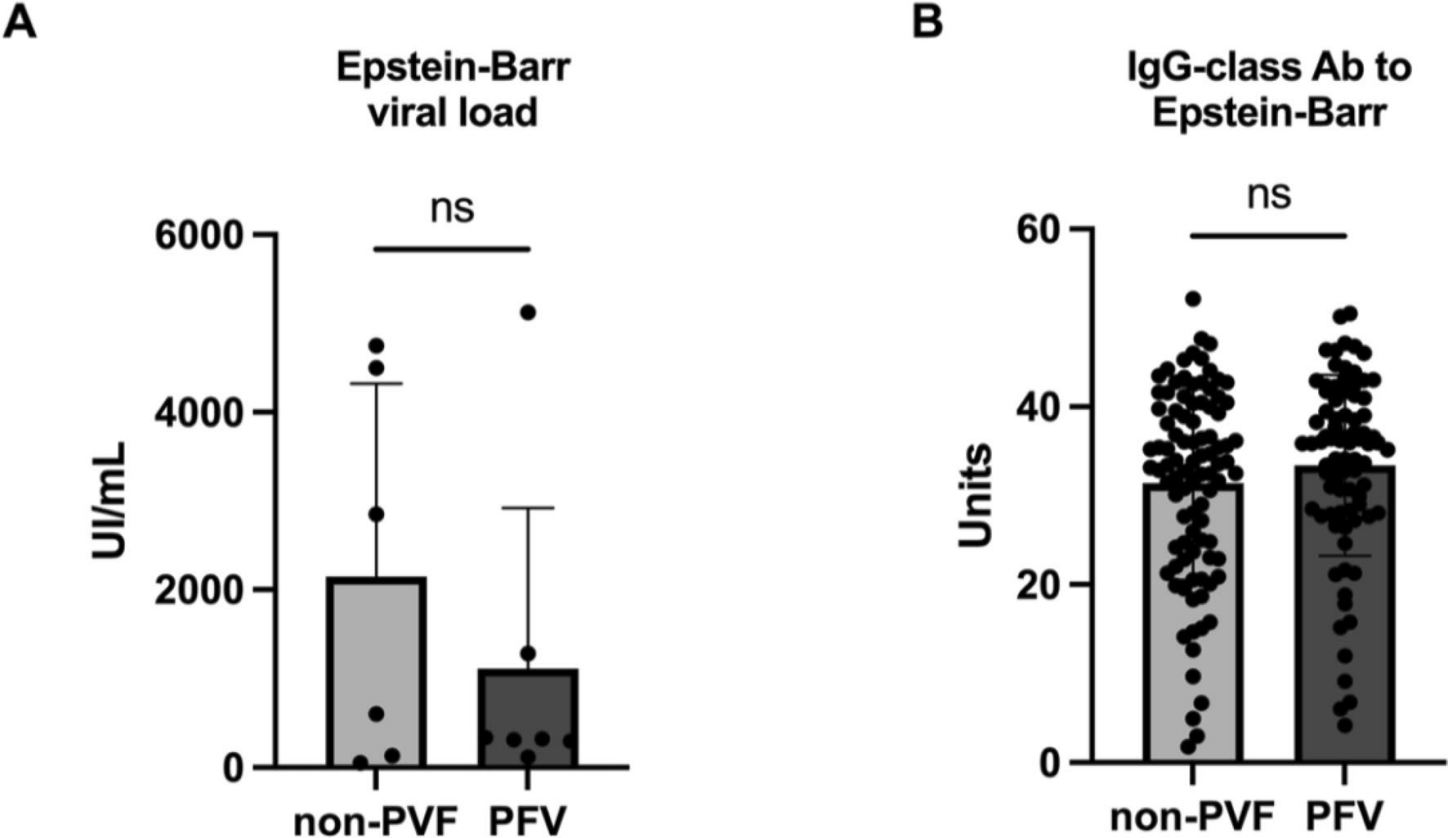
(**A**) Epstein–Barr viral load (UI/mL) and (**B**) IgG-class antibodies to Epstein–Barr nuclear antigen levels (units) in non-PVF patients and PVF patients.

*Human gammaherpesvirus 4* (NC_007605.1) viral load of 15 patients was evaluated by RT-PCR. Viral load was detected in 13 patients and no significant differences were observed between non-PVF and PVF patients (2147 ± 887.2 vs. 1113 ± 683.6 UI/mL; *P* = 0.731) (Fig. 5A).

IgG-class antibodies to Epstein–Barr nuclear antigen were detected in 94.8% of the analyzed population. Similar to the non-significant differences found in Human *gammaherpesvirus 4* (NC_007605.1) frequencies between non-PVF and PVF patients by sequencing, IgG-class antibody detection levels did not significantly differ between non-PVF and PVF patients (31.42 ± 1.121 vs. 33.40 ± 1.125 Units; *P* = 0.196) (Fig. 5B).

Supplementary Table 8 details the relative frequencies of the rest of the identified OTUs. Among the less common OTUs, the *Alphatorquevirus* genus (Fig. 3B), belonging to the *Anelloviridae* family, was most prominently represented, as we identified the complete genome or specific genes of up to 27 *Torque teno virus*, also referred to as transfusion transmitted viruses or TTVs. PVF and non-PVF patients showed significantly different frequencies of *Torque teno virus* 18 (NC_043414.1) and *Torque teno virus* 8 (NC_014084.1). The relative frequency of *Torque teno virus* 18 was significantly higher in non-PVF patients than in PVF patients (0.00058 ± 0.0002 vs. 0.00013 ± 0.00006; *P* = 0.0297) (Fig. 4D). Similarly, the relative frequency of *Torque teno virus* 8 was significantly increased in non-PVF patients compared to PVF patients (0.00038 ± 0.00011 vs. 0.00004 ± 0.00003; *P* = 0.0097) (Fig. 4E).

### Systemic inflammation

**Figure 6:**
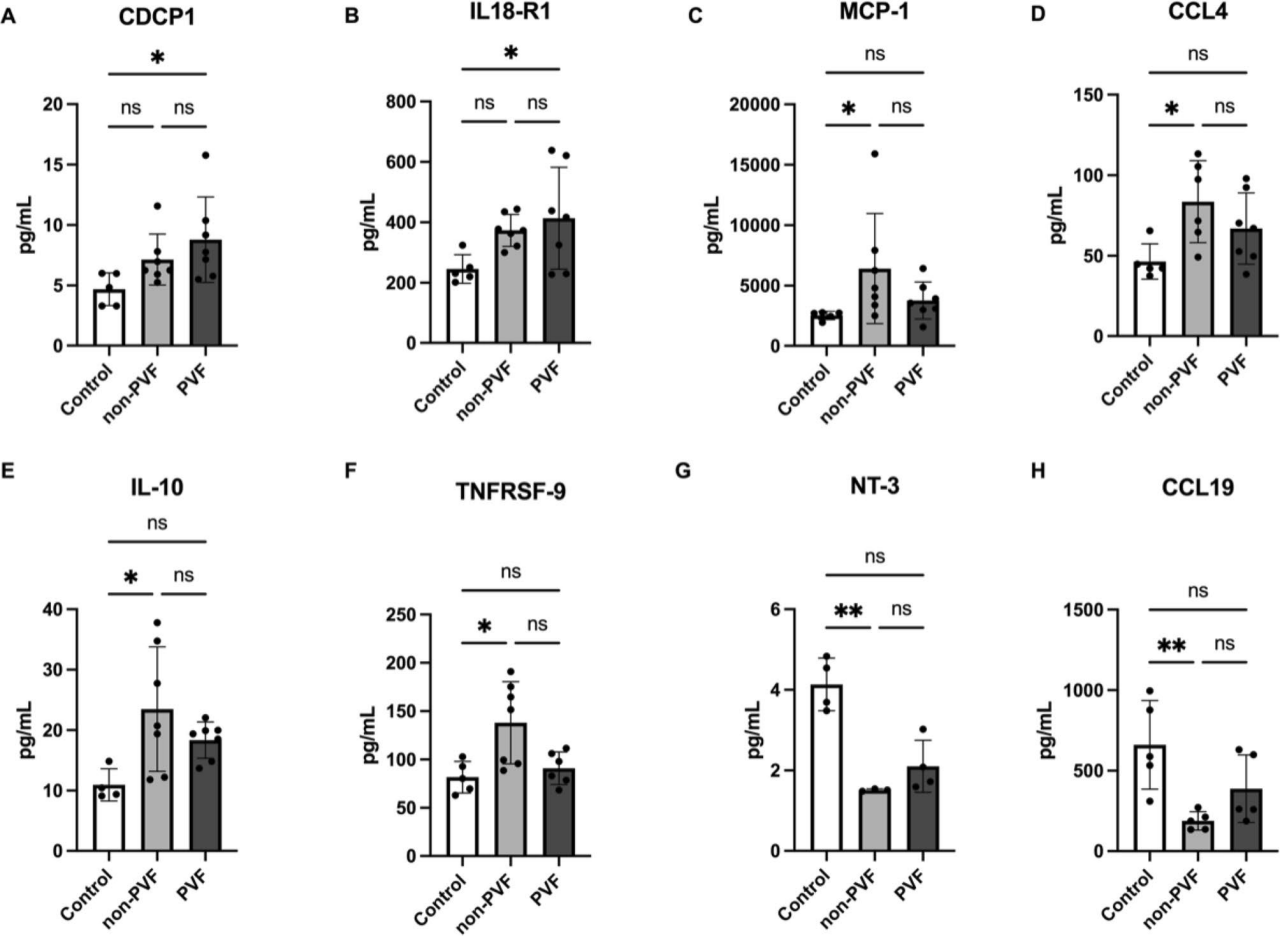
Protein levels (pg/mL) of (**A**) CUB domain containing protein 1 (CDCP1), (**B**) Interleukin-18 receptor 1 (IL18-R1), (**C**) Monocyte chemotactic protein 1 (MCP-1), (**D**) C–C motif chemokine 4 (CCL4), (**E**) Interleukin 10 (IL-10), (**F**) Tumor necrosis factor receptor superfamily member 9 (TNFRSF-9), (**G**) Neurotrophin-3 (NT-3), and (**H**) C–C motif chemokine 19 (CCL19) in healthy subjects, non-PVF patients, and PVF patients. *P < 0.05; **P < 0.01.

The inflammatory proteomic profile was analysed using the Olink Inflammation panel (Supplementary Table 9). Our results did not show that PVF and non-PVF patients significantly differed in any of the analysed inflammatory-related proteins (Table 3). Indeed, 53 of the 92 analysed proteins showed no differences between any of the studied groups (Supplementary Figs. 1, 2, 3 and 4). On the other hand, 16 of the 92 proteins significantly differed between healthy subjects versus both non-PVF and PVF patients (Supplementary Fig. 5). No differences were found between non-PVF and PVF patients; however, some proteins significantly differed between the healthy control group and one of the AMI groups. Compared to healthy subjects, PVF patients showed significantly higher circulating levels of CUB domain containing protein 1 (CDCP1) (*P* = 0.0364) (Fig. 6A) and Interleukin-18 receptor 1 (IL18-R1) (*P* = 0.0488) (Fig. 6B).

**Table 3:** Inflammatory-related protein values in healthy subjects and non-PVF and PVF patients.

	Control	non-PVF	PVF		Control	non-PVF	PVF
CDCP1	4.69 ± 0.60	7.15 ± 0.80	8.79 ± 1.34	IL10RB	68.41 ± 7.43	84.77 ± 9.01	78.31 ± 3.71
IL8	26.30 ± 3.36	99.22 ± 11.69	109.1 ± 13.94	IL18R1	245.7 ± 21.28	373.5 ± 20.01	413.8 ± 63.71
VEGFA	1898 ± 239.40	2239 ± 171.1	1861 ± 151.1	PDL1	41.86 ± 6.62	47.13 ± 3.89	38.75 ± 2.48
CD8A	832.40 ± 265.80	451.2 ± 66.89	598.6 ± 115.5	CXCL5	1919 ± 742	3756 ± 1186	3857 ± 908
MCP3	3.58 ± 0.31	21.31 ± 3.18	15.01 ± 2.01	TRANCE	22.83 ± 3.92	10.97 ± 0.73	8.79 ± 0.72
GDNF	4.11 ± 0.38	1.86 ± 0.37	1.76 ± 0.28	HGF	296.5 ± 41.88	231.7 ± 12.98	677.4 ± 203.2
CD244	68.93 ± 6.30	62.25 ± 12.45	63.34 ± 2.81	IL12B	54.03 ± 5.58	54.54 ± 12.94	48.14 ± 10.19
IL7	3.06 ± 0.57	1.87 ± 0.17	1.94 ± 0.15	MMP10	442.6 ± 112.3	931.2 ± 153.9	799.8 ± 171.6
OPG	1148 ± 80.38	1271 ± 92.98	1496 ± 275.6	IL10	10.95 ± 1.33	23.48 ± 3.89	18.34 ± 1.14
LAP TGFbeta1	65.40 ± 7.80	90.92 ± 15.1	80.97 ± 9.54	TNF	5.43 ± 0.45	5.93 ± 1.02	5.99 ± 1.33
uPA	961.70 ± 93.32	698.3 ± 63.69	748.3 ± 78.62	CCL23	1286 ± 82.81	1829 ± 218.6	2080 ± 331.8
IL6	3.48 ± 0.58	34.48 ± 3.13	37.08 ± 10.22	CD5	41.45 ± 3.77	46.65 ± 3.28	39.92 ± 1.75
IL17C	6.25 ± 1.17	7.56 ± 2.41	10.96 ± 4.86	CCL3	36.42 ± 3.64	66.64 ± 11.59	51.1 ± 6.46
MCP1	2521 ± 157.20	6408 ± 1724	3771 ± 581.9	FIt3L	555.4 ± 52.14	286.4 ± 35.88	247.6 ± 21.49
IL17A	3.72 ± 1.34	2.74 ± 0.51	2.304 ± 0.39	CXCL6	284.6 ± 86.37	430.2 ± 38.51	489 ± 35.34
CXCL11	164.7 ± 25.89	911.2 ± 93.37	866.3 ± 66.89	CXCL10	791 ± 187.2	469.4 ± 58.07	420.7 ± 65.33
AXIN1	35.7 ± 7.77	8.99 ± 2.74	5.37 ± 0.98	4EBP1	632.1 ± 150.8	131.6 ± 43.74	404.7 ± 174.6
TRAIL	202 ± 20.69	106.1 ± 13.91	95.39 ± 9.25	SIRT2	44.72 ± 17.29	24.32 ± 6.24	15.30 ± 3.48
CXCL9	93.99 ± 7.32	162.3 ± 24.21	171.5 ± 24.54	CCL28	3.89 ± 0.36	3.09 ± 0.303	3.019 ± 0.25
CST5	90.25 ± 17.83	129.6 ± 31.99	102.3 ± 18.42	DNER	388.9 ± 13.48	366.5 ± 25.61	381.9 ± 15.06
OSM	30.11 ± 5.66	19.79 ± 1.96	17.74 ± 2.78	ENRAGE	4.96 ± 0.51	18.07 ± 1.46	18.13 ± 4.77
CXCL1	863.6 ± 315.90	1056 ± 226.7	1137 ± 113.3	CD40	3126 ± 361.80	3528 ± 701.7	2548 ± 164.3
CCL4	46.48 ± 4.90	83.61 ± 10.38	66.94 ± 8.35	IFNgamma	112.3 ± 21	48.86 ± 12.09	70.59 ± 25.32
CD6	60.99 ± 6.57	43.34 ± 5.53	49.95 ± 6.49	FGF19	609.4 ± 118.1	513.3 ± 105.1	290.2 ± 52.22
SCF	640.10 ± 73.65	664.8 ± 111.8	612.4 ± 78.06	LIF	0.96 ± 0	1.29 ± 0.16	1.29 ± 0.09
IL18	447.7 ± 65.92	489.1 ± 62.24	422.8 ± 30.51	MCP2	349.9 ± 47.23	298.5 ± 37.01	375.3 ± 51.28
SLAMF1	3.81 ± 0.50	4.54 ± 0.57	3.5 ± 0.25	CASP8	4.97 ± 0.71	7.09 ± 1.28	5.89 ± 0.36
TGFalpha	6.19 ± 0.44	7.83 ± 0.77	7.92 ± 1.41	CCL25	86.89 ± 6.94	63.32 ± 5.83	77.15 ± 11.5
MCP4	16,253 ± 3264	81,152 ± 19,819	50,896 ± 3428	CX3CL1	14.37 ± 2.56	26.04 ± 3.69	22.64 ± 2.91
CCL11	230.40 ± 24.26	481.4 ± 33.95	381.5 ± 24.58	TNFRSF9	81.7 ± 7.30	138.1 ± 16.04	90.95 ± 6.88
TNFSF14	19.30 ± 2.67	28.12 ± 7.01	21.84 ± 3.30	NT3	4.14 ± 0.33	1.51 ± 0.02	2.10 ± 0.32
FGF23	3.12 ± 0.16	3.61 ± 0.51	3.58 ± 0.49	TWEAK	459.6 ± 22.5	313.9 ± 14.53	265.8 ± 30.13
FGF5	1.86 ± 0.06	1.74 ± 0.07	1.70 ± 0.11	CCL20	220.4 ± 57.35	328.5 ± 72.55	214.7 ± 49.73
MMP1	12,364 ± 2748	16,107 ± 3805	16,403 ± 5581	ST1A1	8.048 ± 2.26	17.51 ± 4.81	6.23 ± 1.07
LIFR	13.83 ± 1.64	18.22 ± 1.59	17.35 ± 0.96	STAMBP	74.1 ± 29.24	31.81 ± 8.30	23.75 ± 4.36
FGF21	59.42 ± 18.94	60.01 ± 13.57	153.9 ± 55.97	ADA	46.35 ± 7.53	37.58 ± 2.41	38.06 ± 6.38
CCL19	660.8 ± 123	188.4 ± 25.76	387.9 ± 93.94	TNFB	22.2 ± 1.57	14.86 ± 2.21	12.72 ± 1.56
IL15RA	2.02 ± 0.11	2.19 ± 0.25	2.02 ± 0.14	CSF1	1009 ± 106.9	1506 ± 86.57	1404 ± 81.83

Monocyte chemotactic protein 1 (MCP-1), C–C motif chemokine 4 (CCL4), Tumor necrosis factor receptor superfamily member 9 (TNFRSF-9), Interleukin-10 (IL-10), Chemokine (C–C motif) ligand 19 (CCL19), and Neurotrophin-3 (NT-3) each showed a different expression profile between healthy subjects and non-PVF patients, but did not exhibit significant differences when compared with PVF patients. MCP1 (*P* = 0.0221) (Fig. 6C), CCL4 (*P* = 0.0281) (Fig. 6D), IL-10 (*P* = 0.0279) (Fig. 6E), and TNFRSF-9 (*P* = 0.0499) (Fig. 6F) levels were significantly promoted in non-PVF patients in comparison with the control groups. Conversely, NT-3 (*P* = 0.0092) (Fig. 6G) and CCL19 (*P* = 0.0093) (Fig. 6H) levels were significantly lower in non-PVF patients compared with in healthy subjects.

## Discussion

Primary ventricular fibrillation (PVF) is among the leading causes of prehospital sudden cardiac death. It is presently unknown what factors increase the probability of PVF development during acute ischemia, complicating the identification of PVF predictors. We thus aimed to evaluate possible PVF predictors or triggers, including the complete DNA virome and the inflammatory proteome in PPCI-treated STEMI patients.

A growing number of viruses have been determined to be associated with inflammatory cardiomyopathy. Previous data suggest that viral exposure could increase PVF susceptibility, although this has not been conclusively proven. In this context, Andréoletti et al. identified coxsackievirus B infection in post-mortem endomyocardial tissue of patients who died suddenly due to AMI^10^. Additionally, the AGNES (Arrhythmia Genetics in the NEtherlandS) study showed that PVF during first STEMI was most significantly associated with SNP rs2824292 at chromosome 21q21, where the *CXADR* gene is found. *CXADR* encodes the coxsackie and adenovirus receptor protein, which has been implicated in myocarditis^28^, dilated cardiomyopathy^28^, and ventricular conduction and arrhythmia vulnerability^29^. However, this association was not replicated in at least two additional studies^30,31^. Extreme influenza epidemics are also reportedly associated with out-of-hospital cardiac arrest^8^. However, no other relationships have been found between PVF occurrence and enterovirus or influenza exposure^9^.

The present pilot study is the first to include a circulating virome analysis of all DNA viruses that infect vertebrates. Our findings indicate that non-PVF and PVF patients significantly differed only in the levels of *Macacine gammaherpesvirus 4* (Rhesus lymphocryptovirus)*, Human herpesvirus 4 type 2* (Epstein–Barr virus type 2)*,* and *Torque teno viruses 8* and *18* (transfusion transmitted viruses).

Gamma-herpesvirinae family viruses are lymphotropic viruses that infect lymphoid cells. Epstein–Barr virus (EBV) is a highly ubiquitous herpesvirus which asymptomatically infect over 90% of the population^32^. Once infected, EBV persists in B-cells for life and could be reactivated in immunosuppression cases^33^. In terms of the heart, EBV reportedly induces severe infection of T-cells in the myocardium of patients with ongoing myopericarditis^34,35^, as well as in abdominal or coronary aneurysms^36,37^. EBV infection may also influence the development of atherosclerosis^38^. Here we identified EBV (*Human gammaherpesvirus 4*) and EBV type 2 (*Human herpesvirus 4 type 2*). The relative frequency of EBV did not significantly differ between non-PVF and PVF patients. Along this line, we did not find significant differences in the viral load or in the IgG-class antibodies to EBV between non-PVF and PVF patients measured by RT-PCR and ELISA, respectively. On the other hand, the EBV type 2 frequency was significantly higher in non-PVF patients than in PVF patients, and is thus not a risk factor for second-hit ischaemia-driven cardiac arrest. Any of the patients analysed took immunosuppressive treatment or had any malignancy.

Furthermore, Torque teno viruses (TTVs) are small DNA viruses that have been detected in many mammalian hosts, and whose prevalence in humans is > 90%^39^. It is not clear that TTVs act as primary pathogens, and it appears that TTVs usually establish chronic infections without causing pathology. It has been suggested that TTVs could be used as markers of viral environmental contamination, since TTVs are potential contaminants in water sources^40^ and hospitals^41^, including in the blood supply^42^. This may explain why we detected 27 species of TTVs in the presented study. Remarkably, among 20 human samples, only 1 tested negative for all detected TTV species. Although they are not among the 10 most frequent relative entries, TTV-8 and TTV-18 were the most frequently detected TTVs, and their frequencies significantly differed between non-PVF and PVF patients. However, the relative frequencies of TTV-8 and TTV-18 were significantly higher in non-PVF patients than in PVF patients, and thus do not provide information to predict sudden cardiac arrest. Takeuchi et al. detected one TTV sequence read in a patient with acute myocarditis, but could not establish it as a potential pathogen of myocarditis^43^. Both our results and Takeuchi’s findings support the widespread idea that TTVs are unlikely to act as primary pathogens.

The second objective of this study was to examine the systemic inflammatory response, which is known to play important roles in the pathophysiology of acute coronary syndrome and atherosclerosis. Notably, in recent years, its involvement in SCD has also been studied, although attempts to find predictive biomarkers have yielded inconclusive results^44^. The Physicians’ Health Study showed that C-reactive protein (CRP) levels are an independent risk factor for SCD (OR, 2.78; 95% CI, 1.35–5.72)^45^. In contrast, the Nurses’ Health Study did not confirm any significant correlation between SCD and highly sensitive CRP^46^. Among healthy European middle-aged men who participated in the PRIME Study, higher IL-6 was a strong predictor of sudden death, with an OR of 3.06 (95% CI, 1.20–7.81)^11^, but CRP was not shown to predict SCD, as in the Nurses’ Health Study. Furthermore, our group identified growth differentiation factor 15 (GDF-15) as a predictor of mortality and CV morbidity^47^, and Andersson et al. detected GDF-15 as a risk factor for sudden cardiac death in the acute phase of MI, with an OR of 1.47 (95% CI, 1.11–1.95)^12^.

Our analyses revealed no significant differences between non-PVF and PVF patients for any of the analysed inflammatory-related proteins. We did identify differential protein expression between healthy subjects and STEMI patients (including both non-PVF and PVF patients) (Supplementary Fig. 5). Compared to healthy subjects, STEMI patients showed significantly higher levels of inflammatory proteins related to cell adhesion, chemotaxis, and cellular response to cytokine stimulus, and cell activation proteins involved in immune response, such as IL-6, IL-8 CXCL11, CCL11, MCP3, MCP4, and ENRAGE. The roles of IL-6 and IL-8 in AMI have been previously described^48,49^. CCL11 has potent eosinophil chemoattractant activity, and is expressed by cardiac macrophages^50^. Here we found that CCL11 levels were increased in STEMI patients compared to healthy subjects, thus confirming the previously observed association between CCL11 and myocardial infarction^51,52^. MCP-3 plays an important role in cell recruitment to inflammatory sites, specifically, it has been described that MCP-3 recruits mesenchymal stem cells and improved cardiac remodeling^53^. Mao et al., found that MCP-3 levels were decreased in patients with cardiac remodeling after AMI compared to MI and control groups; in addition, MCP-3 values were not differential between MI and healthy subjects^54^. These results do not agree with what was found in our pilot study, so delving into the role that MCP-3 plays in STEMI patients would be interesting.

Although no differences were found between non-PVF and PVF patients, some proteins significantly differed between the healthy control group and one of the STEMI groups. For example, CDCP1 and IL18-R1 were significantly higher in PVF patients than in healthy subjects. Shia et al. conducted genome-wide association analyses, and identified variations in the DNA sequence that affect the expression of 3p21.31 (CDCP1), which were associated with myocardial infarction^55^. Those authors did not specify whether the patients had PVF. In the other hand, Ponasenko et al. also found that a polymorphic variant of *IL18R1* was associated with an increased risk of MI in CAD patients with coronary artery disease^56^. Based on our results, it would be interesting to further examine into the studies related to CDCP1 or IL18-R1 and PVF. In contrast, MCP-1, CCL4, TNFRSF-9, and NT-3 showed different expression profiles in healthy subjects compared to non-FVP patients, but not compared with FVP patients. The association of some of them with cardiovascular disorders has already been previously described by other authors. MCP-1, which recruit circulating monocytes, plays a major role in the immunologic profile of ischaemia/reperfusion injury in the heart^57^; CCL4 is directly involved in the atheroma plaque stabilization^58^; and elevated NT-3 plasma levels are associated with an increased risk atrial fibrillation recurrence^59^. However, there remains a need to elucidate potential key roles of these proteins in inflammatory process development in AMI; and they do not seem to be involved in PVF.

This study has several limitations. It was a pilot study with a limited sample size. Despite comprehensive examination of both the virome and the proteome, we did not identify any clear trend. The VirCapSeq-VERT panel can capture both DNA and RNA viruses; however, due to the storage conditions and available blood material, we cannot fully exclude the presence of undetected RNA viruses. In addition, we have not been able to make the correlation between the OTUs and the inflammatory protein levels because, although the population is the same, some samples were used to the virome screening study and others to the inflammation analyses. Lastly, to confirm the presence of a viral genome within the myocardium during the acute phase of STEMI, we would need to perform endomyocardial biopsies, which is ethically unacceptable.

In conclusion, our observations revealed no clear trend in associations between the circulating virome or inflammatory proteome and PVF in STEMI. Hence, there remains a critical need for new strategies to better elucidate the possible triggers of PVF, and to identify individuals at high risk of SCD.

## Supplementary Information

Supplementary Figures.

Supplementary Tables.

## Data Availability

The datasets generated during and/or analysed during the current study are available from the corresponding author on reasonable request.
