# Supplementary material for: Circulating virome and inflammatory proteome in patients with ST-elevation myocardial infarction and primary ventricular fibrillation
Source: Sci Rep. 2022 May 12;12:7910. doi: 10.1038/s41598-022-12075-x (PMC9098642; doi:10.1038/s41598-022-12075-x)

**SUPPLEMENTARY FIGURE LEGENDS**

**Supplementary Figure 1.** Protein levels (pg/mL) of T-cell surface glycoprotein CD8 alpha chain (CD8A), Vascular endothelial growth factor A (VEGF), Natural killer cell receptor 2B4 (CD244), Osteoprotegerin (OPG), Transforming growth factor beta-1 proprotein (LAP TGFbeta1), Urokinase-type plasminogen activator (uPA), Interleukin-17A (IL17A), C-X-C motif chemokine 9 (CXCL9), Cystatin-D (CST5), Oncostatin-M (OSM), Growth-regulated alpha protein (CXCL1), T-cell differentiation antigen CD6 (CD6), Kit ligand (SCF), Interleukin-18 (IL-18), Signaling lymphocytic activation molecule (SLAMF1), and Transforming growth factor alpha (TGFalpha) in healthy subjects, non-PVF patients, and PVF patients.

**Supplementary Figure 2.** Protein levels (pg/mL) of Tumor necrosis factor ligand superfamily member 14 (TNFSF14), Fibroblast growth factor 23 (FGF23), Fibroblast growth factor 5 (FGF5), Interstitial collagenase (MMP1), Leukemia inhibitory factor receptor (LIFR), Fibroblast growth factor 21 (FGF21), Interleukin-15 receptor subunit alpha (IL15RA), Interleukin-10 receptor subunit beta (IL10RB), Programmed cell death 1 ligand 1 (PDL1), C-X-C motif chemokine 5 (CXCL5), Hepatocyte growth factor (HGF), Interleukin-12 subunit beta (IL12B), Stromelysin-2 (MMP10), Tumor necrosis factor (TNF), C-C motif chemokine 23 (CCL23), and T-cell surface glycoprotein CD5 (CD5) in healthy subjects, non-PVF patients, and PVF patients.

**Supplementary Figure 3.** Protein levels (pg/mL) of C-C motif chemokine 3 (CCL3), C-X-C motif chemokine 6 (CXCL6), C-X-C motif chemokine 10 (CXCL10), Eukaryotic translation initiation factor 4E-binding protein 1 (4E-BP1), NAD-dependent protein deacetylase sirtuin-2 (SIRT2), C-C motif chemokine 28 (CCL28), Delta and Notch-like epidermal growth factor-related receptor (DNER), Tumor necrosis factor receptor superfamily member 5 (CD40), Interferon gamma (IFN-gamma), Fibroblast growth factor 19 (FGF19), Leukemia inhibitory factor (LIF), C-C motif chemokine 8 (MCP2), Caspase-8 (CASP8), C-C motif chemokine 25 (CCL25), Fractalkine (CX3CL1), and C-C motif chemokine 20 (CCL20) in healthy subjects, non-PVF patients, and PVF patients.

**Supplementary Figure 4.** Protein levels (pg/mL) of Sulfotransferase 1A1 (ST1A1), STAM Binding Protein (STAMBP), Adenosine deaminase (ADA), and Interleukin-17C (IL17C) in healthy subjects, non-PVF patients, and PVF patients.

**Supplementary Figure 5.** Protein levels (pg/mL) of Interleukin-8 (IL8), C-C motif chemokine 7 (MCP-3), Glial cell line-derived neurotrophic factor (GDNF), Interleukin-7 (IL7), Interleukin-6 (IL6), C-X-C motif chemokine 11 (CXCL11), Axin-1 (AXIN1), Tumor necrosis factor ligand superfamily member 10 (TRAIL), C-C motif chemokine 13 (MCP-4), Eotaxin (CCL11), Tumor necrosis factor ligand superfamily member 11 (TRANCE), Fms-related tyrosine kinase 3 ligand (Fit3L), Protein S100-A12 (ENRAGE), Tumor necrosis factor-like weak inducer of apoptosis (TWEAK), Lymphotoxin-alpha (TNFB), and Macrophage colony-stimulating factor 1 (CSF1). * *P* < 0.05; ** *P* < 0.01; *** *P* < 0.001; **** *P* < 0.0001.


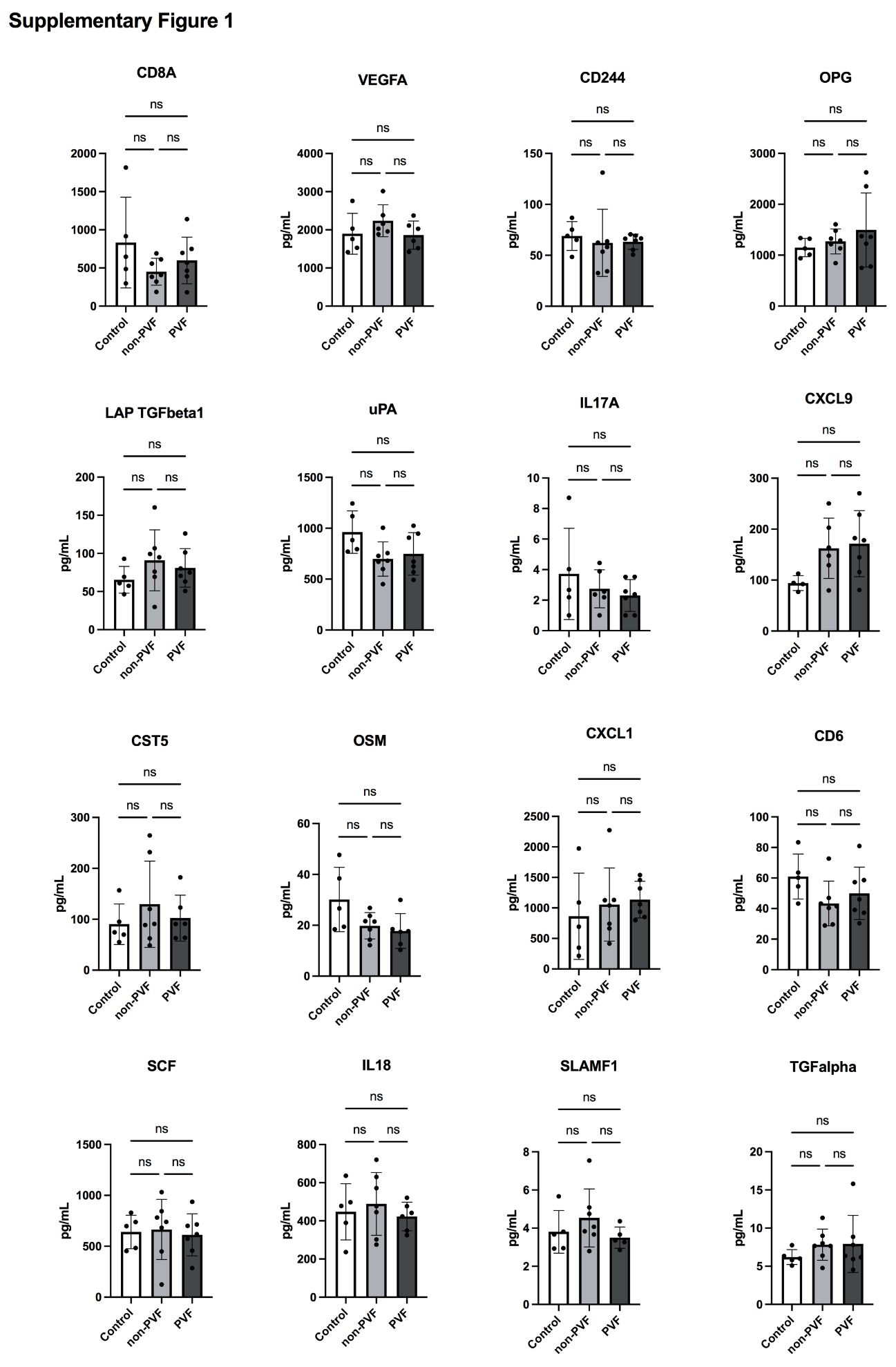


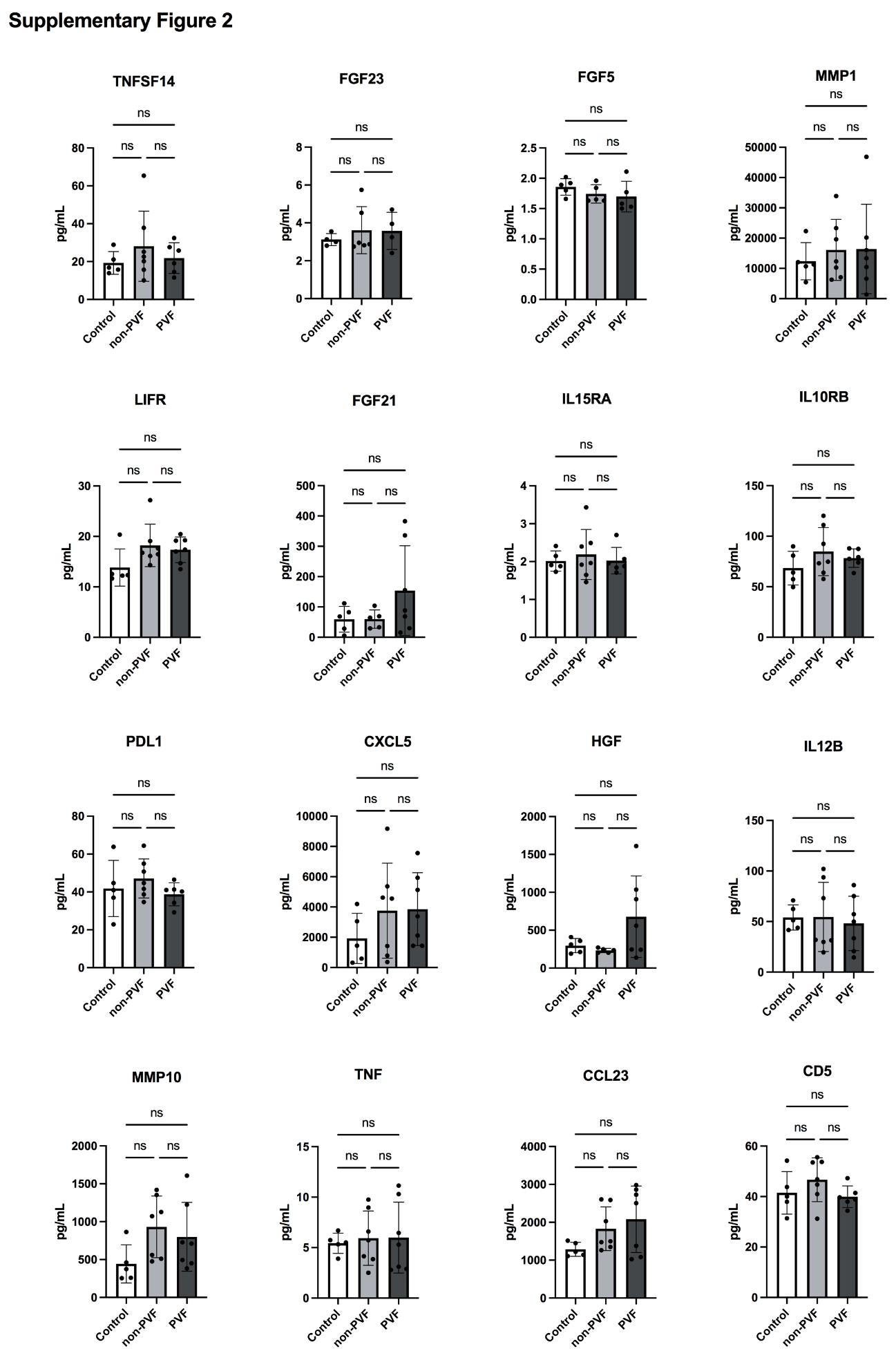


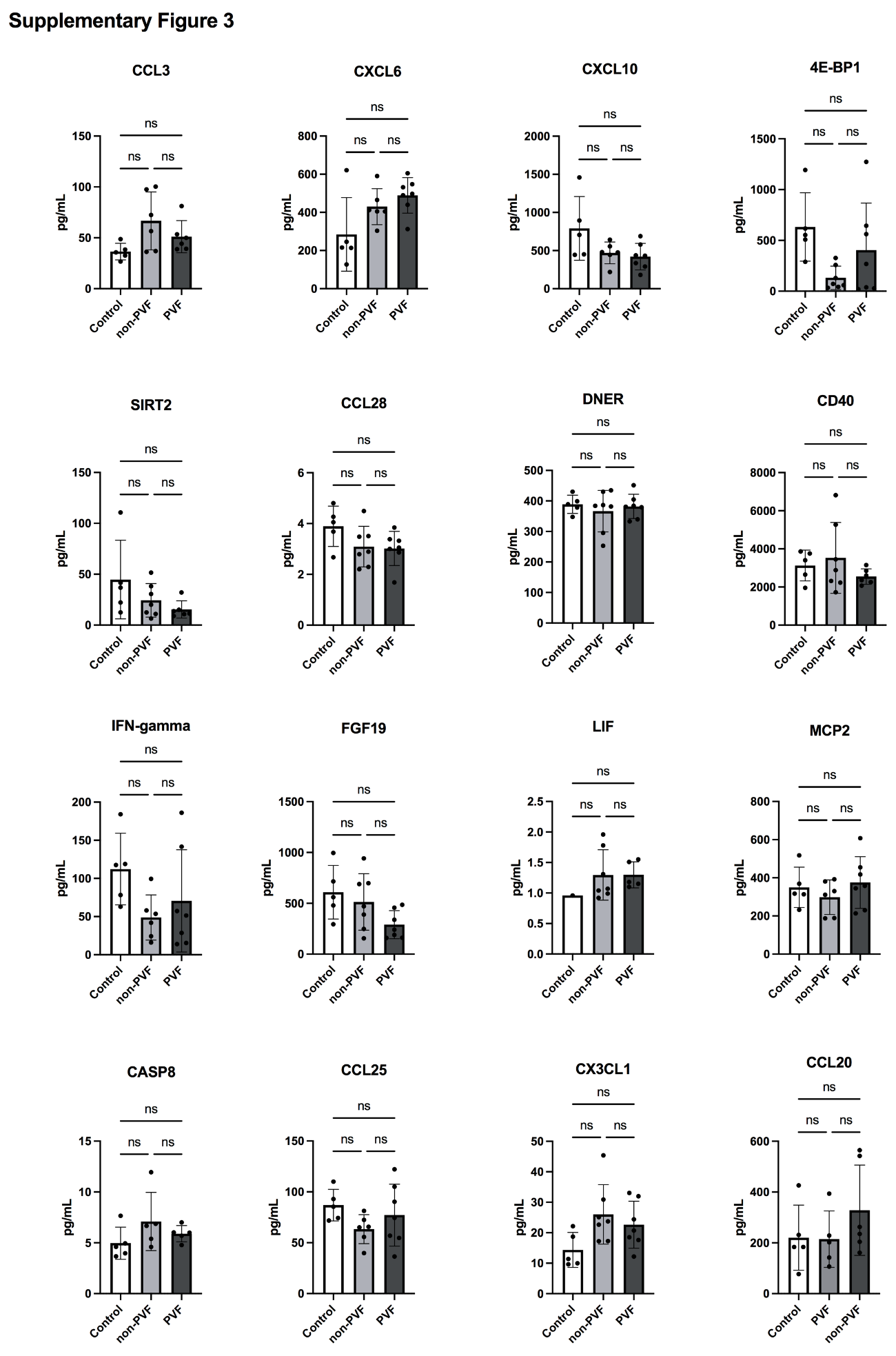


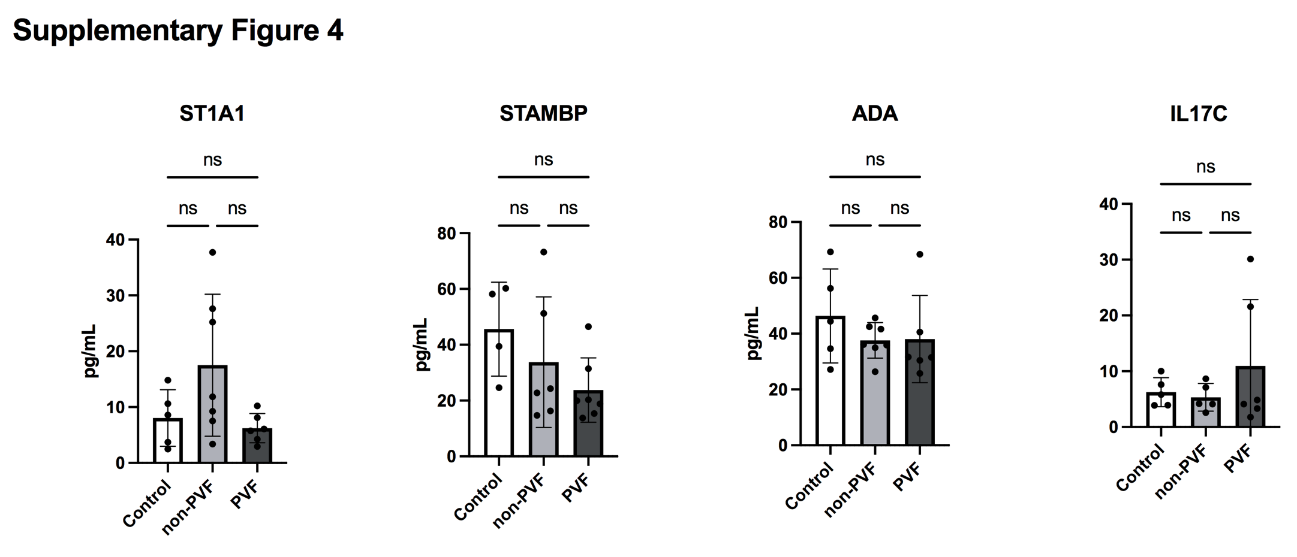


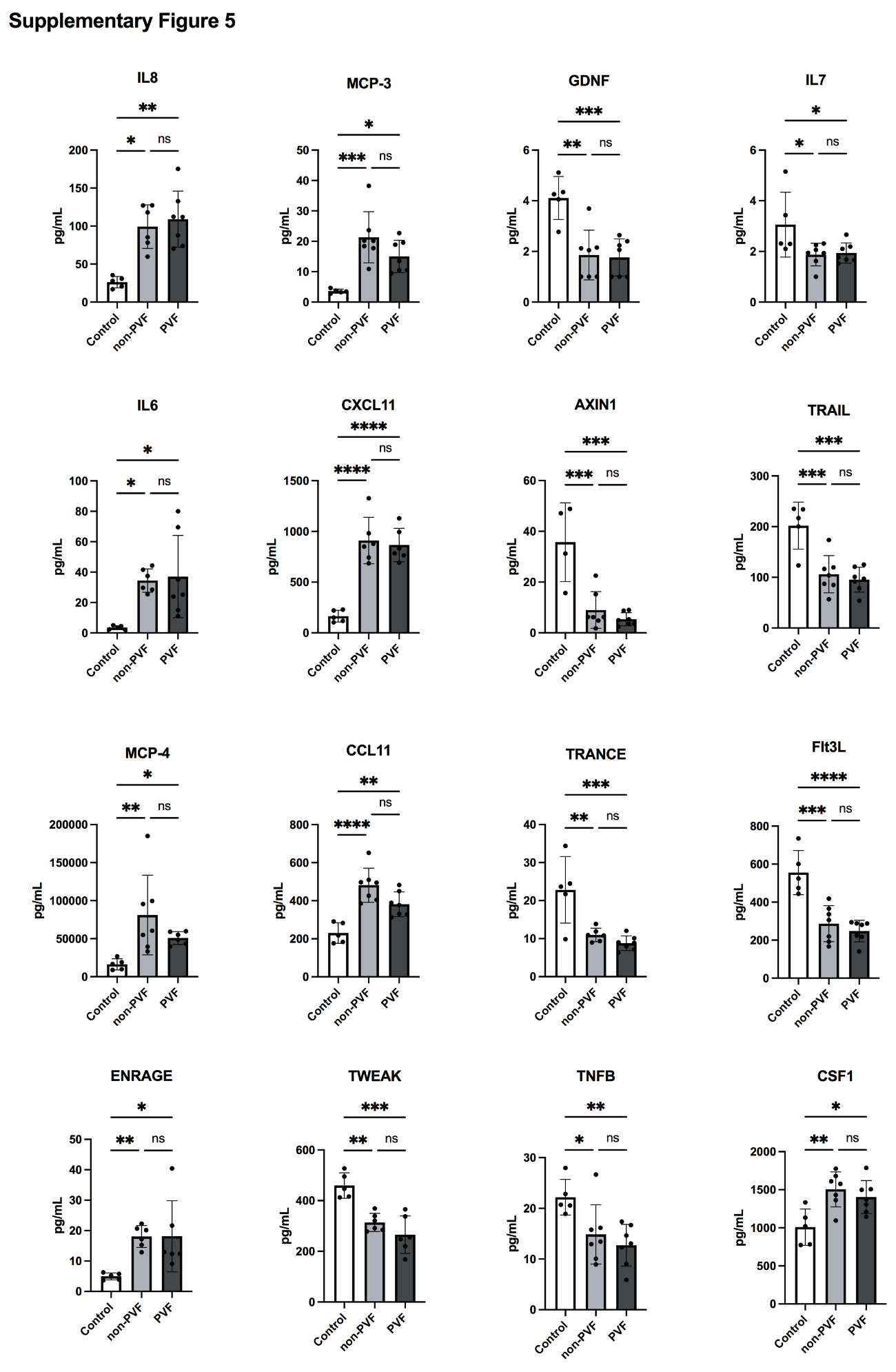

Supplement: Supplementary file 1 — Supplementary Figures. [file 41598_2022_12075_MOESM1_ESM.docx]
