# Supplementary material for: Circulating virome and inflammatory proteome in patients with ST-elevation myocardial infarction and primary ventricular fibrillation
Source: Sci Rep. 2022 May 12;12:7910. doi: 10.1038/s41598-022-12075-x (PMC9098642; doi:10.1038/s41598-022-12075-x)
Supplement: Supplementary file 2 — Supplementary Tables. [file 41598_2022_12075_MOESM2_ESM.docx]

**Supplementary Table 1.** Host depletion statistics.

|  | **Name** | **Total Sequences** | ***Homo sapiens*** | ***%*** | **Host depleted reads** | ***%*** |
| --- | --- | --- | --- | --- | --- | --- |
| **non-PVF** | sample_118o | 10558442 | 10370284 | 98.22 | **184751** | 1.75 |
|  | sample_118r | 10106008 | 9885574 | 97.82 | **162394** | 1.61 |
|  | sample_119v | 11048770 | 10857645 | 98.27 | **181196** | 1.64 |
|  | sample_121d | 33884809 | 33144398 | 97.81 | **543297** | 1.60 |
|  | sample_121p | 9920662 | 9758092 | 98.36 | **151585** | 1.53 |
|  | sample_122g | 11165023 | 10943967 | 98.02 | **200941** | 1.80 |
|  | sample_123k | 10864662 | 10665162 | 98.16 | **171347** | 1.58 |
|  | sample_124w | 10465597 | 10276540 | 98.19 | **173621** | 1.66 |
|  | sample_126f | 12154970 | 11923783 | 98.10 | **217968** | 1.79 |
| **PVF** | sample_118z | 10463593 | 10271796 | 98.17 | **178899** | 1.71 |
|  | sample_119l | 8648035 | 8496149 | 98.24 | **148323** | 1.72 |
|  | sample_120s | 10897938 | 10675637 | 97.96 | **195539** | 1.79 |
|  | sample_121i | 8408035 | 8245611 | 98.07 | **149350** | 1.78 |
|  | sample_121j | 8359848 | 8181474 | 97.87 | **160291** | 1.92 |
|  | sample_123u | 10103203 | 9900356 | 97.99 | **180584** | 1.79 |
|  | sample_124c | 8886910 | 8712976 | 98.04 | **157783** | 1.78 |
|  | sample_125a | 10242017 | 10067915 | 98.30 | **163697** | 1.60 |
|  | sample_125k | 10663558 | 10480589 | 98.28 | **169373** | 1.59 |
|  | sample_125x | 10833562 | 10645529 | 98.26 | **171977** | 1.59 |
|  | sample_126b | 10852880 | 10656713 | 98.19 | **178591** | 1.65 |

**Supplementary Table 2.** Quality control statistics for raw data.

|  | **Sample Name** | **File names** | **% PCR Duplicates** | **% GC** | **Average Sequence Length** | **Million reads** |
| --- | --- | --- | --- | --- | --- | --- |
| **non-PVF** | sample_118o | sample_118o_R1 | 63.795 | 46 | 101 | 184751 |
|  |  | sample_118o_R2 | 59.160 | 46 | 101 | 184751 |
|  | sample_118r | sample_118r_R1 | 63.534 | 46 | 101 | 162394 |
|  |  | sample_118r_R2 | 59.637 | 46 | 101 | 162394 |
|  | sample_119v | sample_119v_R1 | 63.041 | 46 | 101 | 181196 |
|  |  | sample_119v_R2 | 58.351 | 47 | 101 | 181196 |
|  | sample_121d | sample_121d_R1 | 66.748 | 45 | 101 | 543297 |
|  |  | sample_121d_R2 | 64.233 | 45 | 101 | 543297 |
|  | sample_121p | sample_121p_R1 | 62.316 | 46 | 101 | 151585 |
|  |  | sample_121p_R2 | 56.900 | 46 | 101 | 151585 |
|  | sample_122g | sample_122g_R1 | 62.996 | 46 | 101 | 200941 |
|  |  | sample_122g_R2 | 57.575 | 47 | 101 | 200941 |
|  | sample_123k | sample_123k_R1 | 63.727 | 45 | 101 | 171347 |
|  |  | sample_123k_R2 | 58.949 | 46 | 101 | 171347 |
|  | sample_124w | sample_124w_R1 | 63.818 | 45 | 101 | 173621 |
|  |  | sample_124w_R2 | 58.331 | 46 | 101 | 173621 |
|  | sample_126f | sample_126f_R1 | 63.660 | 46 | 101 | 217968 |
|  |  | sample_126f_R2 | 59.591 | 47 | 101 | 217968 |
| **PVF** | sample_118z | sample_118z_R1 | 64.972 | 45 | 101 | 178899 |
|  |  | sample_118z_R2 | 61.305 | 46 | 101 | 178899 |
|  | sample_119l | sample_119l_R1 | 64.547 | 46 | 101 | 148323 |
|  |  | sample_119l_R2 | 60.949 | 47 | 101 | 148323 |
|  | sample_120s | sample_120s_R1 | 64.706 | 45 | 101 | 195539 |
|  |  | sample_120s_R2 | 61.258 | 46 | 101 | 195539 |
|  | sample_121i | sample_121i_R1 | 63.588 | 46 | 101 | 149350 |
|  |  | sample_121i_R2 | 60.179 | 46 | 101 | 149350 |
|  | sample_121j | sample_121j_R1 | 65.161 | 45 | 101 | 160291 |
|  |  | sample_121j_R2 | 62.518 | 46 | 101 | 160291 |
|  | sample_123u | sample_123u_R1 | 63.705 | 46 | 101 | 180584 |
|  |  | sample_123u_R2 | 60.197 | 47 | 101 | 180584 |
|  | sample_124c | sample_124c_R1 | 64.170 | 46 | 101 | 157783 |
|  |  | sample_124c_R2 | 59.898 | 47 | 101 | 157783 |
|  | sample_125a | sample_125a_R1 | 62.824 | 45 | 101 | 163697 |
|  |  | sample_125a_R2 | 57.862 | 46 | 101 | 163697 |
|  | sample_125k | sample_125k_R1 | 65.247 | 46 | 101 | 169373 |
|  |  | sample_125k_R2 | 61.525 | 46 | 101 | 169373 |
|  | sample_125x | sample_125x_R1 | 61.911 | 46 | 101 | 171977 |
|  |  | sample_125x_R2 | 55.963 | 47 | 101 | 171977 |
|  | sample_126b | sample_126b_R1 | 62.872 | 46 | 101 | 178591 |
|  |  | sample_126b_R2 | 57.790 | 47 | 101 | 178591 |

**Supplementary Table 3**. Trimming statistics.

|  |  |  | **Surviving Reads** | | **Forward Only Surviving** | | **Reverse Only Surviving** | | **Dropped** | |
| --- | --- | --- | --- | --- | --- | --- | --- | --- | --- | --- |
|  | **Sample Name** | **Total** | number | % | number | % | number | % | number | % |
| **non-PVF** | **sample_118o** | 184751 | 109289 | 59.155 | 70910 | 38.381 | 1044 | 0.565 | 3508 | 1.899 |
|  | **sample_118r** | 162394 | 106962 | 65.866 | 52117 | 32.093 | 883 | 0.544 | 2432 | 1.498 |
|  | **sample_119v** | 181196 | 111045 | 61.284 | 65272 | 36.023 | 1184 | 0.653 | 3695 | 2.039 |
|  | **sample_121d** | 543297 | 221381 | 40.748 | 302197 | 55.623 | 3501 | 0.644 | 16218 | 2.985 |
|  | **sample_121p** | 151585 | 99197 | 65.440 | 47558 | 31.374 | 1030 | 0.679 | 3800 | 2.507 |
|  | **sample_122g** | 200941 | 118702 | 59.073 | 77673 | 38.655 | 1127 | 0.561 | 3439 | 1.711 |
|  | **sample_123k** | 171347 | 117379 | 68.504 | 49990 | 29.175 | 972 | 0.567 | 3006 | 1.754 |
|  | **sample_124w** | 173621 | 112581 | 64.843 | 57062 | 32.866 | 989 | 0.570 | 2989 | 1.722 |
|  | **sample_126f** | 217968 | 121690 | 55.829 | 91023 | 41.760 | 1260 | 0.578 | 3995 | 1.833 |
| **PVF** | **sample_118z** | 178899 | 116106 | 64.900 | 59222 | 33.104 | 935 | 0.523 | 2636 | 1.473 |
|  | **sample_119l** | 148323 | 88294 | 59.528 | 56329 | 37.977 | 761 | 0.513 | 2939 | 1.981 |
|  | **sample_120s** | 195539 | 116069 | 59.358 | 75402 | 38.561 | 986 | 0.504 | 3082 | 1.576 |
|  | **sample_121i** | 149350 | 91163 | 61.040 | 52831 | 35.374 | 810 | 0.542 | 4546 | 3.044 |
|  | **sample_121j** | 160291 | 94233 | 58.789 | 61415 | 38.315 | 797 | 0.497 | 3846 | 2.399 |
|  | **sample_123u** | 180584 | 104537 | 57.888 | 71688 | 39.698 | 945 | 0.523 | 3414 | 1.891 |
|  | **sample_124c** | 157783 | 96840 | 61.375 | 57471 | 36.424 | 833 | 0.528 | 2639 | 1.673 |
|  | **sample_125a** | 163697 | 104950 | 64.112 | 54317 | 33.181 | 940 | 0.574 | 3490 | 2.132 |
|  | **sample_125k** | 169373 | 110778 | 65.405 | 55156 | 32.565 | 860 | 0.508 | 2579 | 1.523 |
|  | **sample_125x** | 171977 | 111860 | 65.044 | 55101 | 32.040 | 1146 | 0.666 | 3870 | 2.250 |
|  | **sample_126b** | 178591 | 114978 | 64.381 | 59068 | 33.074 | 1060 | 0.594 | 3485 | 1.951 |

**Supplementary Table 4.** Assembly statistics.

|  |  |  |  | Nucleotides (%) | | | | | | |  |  |
| --- | --- | --- | --- | --- | --- | --- | --- | --- | --- | --- | --- | --- |
|  | Sample Name | Contigs | Bases | A | T | C | G | A+T | C+G | N | Max Length (bp) | N50 (bp) |
| **non-PVF** | sample_118o | 1271 | 199587 | 20.552 | 31.444 | 17.040 | 30.904 | 51.996 | 47.944 | 0.060 | 2783 | 238 |
|  | sample_118r | 1229 | 202818 | 19.763 | 31.749 | 17.437 | 30.894 | 51.512 | 48.331 | 0.158 | 1566 | 246 |
|  | sample_119v | 1361 | 203851 | 19.106 | 31.952 | 16.467 | 32.357 | 51.057 | 48.825 | 0.118 | 1822 | 231 |
|  | sample_121d | 2984 | 436856 | 23.881 | 30.241 | 19.739 | 26.075 | 54.122 | 45.814 | 0.064 | 1610 | 226 |
|  | sample_121p | 985 | 171300 | 19.959 | 31.416 | 17.416 | 31.017 | 51.375 | 48.433 | 0.193 | 1681 | 258 |
|  | sample_122g | 1463 | 220547 | 19.092 | 32.414 | 15.557 | 32.652 | 51.506 | 48.208 | 0.286 | 2130 | 230 |
|  | sample_123k | 1170 | 189259 | 20.613 | 31.539 | 16.609 | 31.112 | 52.152 | 47.721 | 0.127 | 1904 | 249 |
|  | sample_124w | 1199 | 192820 | 19.491 | 32.386 | 15.448 | 32.394 | 51.877 | 47.843 | 0.280 | 1570 | 243 |
|  | sample_126f | 1499 | 215458 | 18.689 | 32.650 | 15.628 | 32.796 | 51.339 | 48.424 | 0.237 | 2155 | 227 |
| **PVF** | sample_118z | 1256 | 204674 | 20.909 | 31.417 | 17.779 | 29.777 | 52.326 | 47.557 | 0.117 | 1983 | 245 |
|  | sample_119l | 1083 | 168810 | 17.511 | 33.215 | 15.961 | 33.313 | 50.726 | 49.274 | 0.000 | 2437 | 230 |
|  | sample_120s | 1237 | 205523 | 20.402 | 31.535 | 17.022 | 30.924 | 51.937 | 47.946 | 0.117 | 1381 | 240 |
|  | sample_121i | 978 | 163278 | 19.607 | 31.611 | 16.602 | 31.795 | 51.218 | 48.397 | 0.386 | 1879 | 250 |
|  | sample_121j | 1166 | 182324 | 20.178 | 31.860 | 16.387 | 31.405 | 52.039 | 47.791 | 0.170 | 1636 | 235 |
|  | sample_123u | 1003 | 183146 | 20.646 | 31.625 | 16.629 | 30.991 | 52.271 | 47.620 | 0.109 | 2293 | 262 |
|  | sample_124c | 1317 | 196722 | 18.455 | 31.773 | 15.281 | 34.378 | 50.228 | 49.660 | 0.112 | 1976 | 233 |
|  | sample_125a | 1306 | 200333 | 20.575 | 30.713 | 17.512 | 30.975 | 51.288 | 48.487 | 0.225 | 2155 | 237 |
|  | sample_125k | 1052 | 186212 | 19.994 | 31.651 | 17.415 | 30.768 | 51.645 | 48.183 | 0.172 | 1697 | 255 |
|  | sample_125x | 1460 | 217450 | 19.128 | 31.256 | 17.179 | 32.184 | 50.384 | 49.364 | 0.253 | 1794 | 236 |
|  | sample_126b | 1503 | 224675 | 20.050 | 30.813 | 16.481 | 32.385 | 50.863 | 48.866 | 0.272 | 1713 | 229 |

**Supplementary Table 5.** Count of non-duplicated reads mapping to each reference sequence and coverage of base pairs (%) of non-PVF patients.

|  |  | **non-FVP** | | | | | | | | | | | | | | | | | |
| --- | --- | --- | --- | --- | --- | --- | --- | --- | --- | --- | --- | --- | --- | --- | --- | --- | --- | --- | --- |
|  |  | sample_118o | | sample_118r | | sample_119v | | sample_121d | | sample_121p | | sample_122g | | sample_123k | | sample_124w | | sample_126f | |
| **Genbank ID** | **Length** | **reads** | **cov (%)** | **reads** | **cov (%)** | **reads** | **cov (%)** | **reads** | **cov (%)** | **reads** | **cov (%)** | **reads** | **cov (%)** | **reads** | **cov (%)** | **reads** | **cov (%)** | **reads** | **cov (%)** |
| **NC_022518.1** | 9472 | 17271 | 83.65 | 18101 | 78.64 | 17594 | 81.28 | 20301 | 89.08 | 16263 | 82.91 | 17978 | 79.55 | 19818 | 79.93 | 19820 | 75.83 | 18822 | 86.21 |
| **NC_001422.1** | 5386 | 27 | 32.84 | 31 | 32.51 | 32 | 43.00 | 138 | 84.72 | 37 | 45.73 | 60 | 58.45 | 45 | 49.33 | 20 | 28.72 | 45 | 54.34 |
| **NC_038336.1** | 3229 | 3 | 4.46 | 2 | 3.07 | 7 | 8.42 | 14 | 21.86 | 0 | 0.00 | 0 | 0.00 | 1 | 0.59 | 0 | 0.00 | 0 | 0.00 |
| **NC_014080.1** | 3736 | 1 | 1.20 | 3 | 4.47 | 3 | 1.71 | 0 | 0.00 | 0 | 0.00 | 0 | 0.00 | 0 | 0.00 | 5 | 3.61 | 0 | 0.00 |
| **NC_014081.1** | 3748 | 4 | 2.99 | 5 | 3.58 | 0 | 0.00 | 16 | 6.32 | 0 | 0.00 | 0 | 0.00 | 0 | 0.00 | 2 | 3.82 | 0 | 0.00 |
| **NC_038340.1** | 3234 | 2 | 3.12 | 8 | 10.30 | 12 | 17.59 | 2 | 1.86 | 1 | 3.12 | 0 | 0.00 | 0 | 0.00 | 0 | 0.00 | 0 | 0.00 |
| **NC_038341.1** | 3153 | 3 | 4.19 | 1 | 1.01 | 0 | 0.00 | 2 | 3.33 | 2 | 3.20 | 0 | 0.00 | 0 | 0.00 | 2 | 1.93 | 0 | 0.00 |
| **NC_014091.1** | 3818 | 8 | 4.71 | 16 | 12.96 | 50 | 54.74 | 69 | 50.79 | 6 | 4.92 | 1 | 0.50 | 0 | 0.00 | 8 | 5.76 | 0 | 0.00 |
| **NC_038339.1** | 3312 | 12 | 22.77 | 15 | 13.29 | 4 | 4.47 | 5 | 6.07 | 10 | 8.85 | 0 | 0.00 | 4 | 4.68 | 2 | 4.02 | 0 | 0.00 |
| **NC_014069.1** | 3690 | 0 | 0.00 | 2 | 1.90 | 0 | 0.00 | 1 | 1.36 | 0 | 0.00 | 0 | 0.00 | 0 | 0.00 | 0 | 0.00 | 0 | 0.00 |
| **NC_001716.2** | 153080 | 954 | 3.29 | 608 | 1.38 | 1474 | 1.59 | 819 | 1.88 | 773 | 1.75 | 956 | 3.58 | 822 | 1.54 | 896 | 1.66 | 1019 | 2.25 |
| **NC_014079.1** | 3798 | 1 | 0.50 | 0 | 0.00 | 0 | 0.00 | 1 | 0.50 | 2 | 0.50 | 3 | 0.63 | 0 | 0.00 | 0 | 0.00 | 1 | 0.50 |
| **NC_014094.1** | 3705 | 1 | 1.46 | 0 | 0.00 | 0 | 0.00 | 4 | 2.13 | 0 | 0.00 | 0 | 0.00 | 0 | 0.00 | 0 | 0.00 | 2 | 0.54 |
| **NC_043414.1** | 3313 | 20 | 18.47 | 33 | 32.93 | 22 | 34.26 | 26 | 22.70 | 7 | 11.47 | 1 | 0.57 | 0 | 0.00 | 0 | 0.00 | 7 | 10.17 |
| **NC_026764.1** | 3774 | 0 | 0.00 | 2 | 0.69 | 1 | 0.74 | 2 | 0.74 | 0 | 0.00 | 0 | 0.00 | 0 | 0.00 | 0 | 0.00 | 3 | 0.69 |
| **NC_026765.1** | 3621 | 7 | 0.55 | 4 | 0.55 | 6 | 0.55 | 13 | 0.55 | 3 | 0.55 | 15 | 0.58 | 5 | 0.55 | 7 | 0.55 | 1 | 0.52 |
| **NC_014074.1** | 3729 | 3 | 0.56 | 2 | 0.56 | 0 | 0.00 | 6 | 4.69 | 1 | 0.56 | 0 | 0.00 | 1 | 0.54 | 0 | 0.00 | 0 | 0.00 |
| **NC_043058.1** | 21614 | 208 | 0.32 | 419 | 0.40 | 739 | 0.32 | 325 | 0.39 | 368 | 0.43 | 317 | 0.40 | 487 | 0.39 | 331 | 0.31 | 568 | 0.31 |
| **NC_032111.1** | 163005 | 544 | 0.20 | 657 | 0.26 | 649 | 0.24 | 1642 | 0.46 | 495 | 0.23 | 662 | 0.23 | 640 | 0.27 | 585 | 0.23 | 957 | 0.25 |
| **NC_009333.1** | 137969 | 12 | 0.08 | 7 | 0.06 | 3 | 0.06 | 15 | 0.12 | 159 | 7.80 | 21 | 0.14 | 7 | 0.05 | 4 | 0.04 | 10 | 0.12 |
| **NC_008168.1** | 104710 | 12 | 0.17 | 15 | 0.18 | 29 | 0.17 | 23 | 0.27 | 14 | 0.13 | 23 | 0.22 | 12 | 0.14 | 19 | 0.15 | 17 | 0.14 |
| **NC_007605.1** | 171823 | 61 | 0.41 | 165 | 4.20 | 98 | 0.10 | 1332 | 34.65 | 73 | 0.30 | 73 | 0.08 | 110 | 1.68 | 89 | 2.06 | 86 | 0.91 |
| **NC_006146.1** | 171096 | 167 | 0.05 | 172 | 0.07 | 182 | 0.05 | 238 | 0.08 | 155 | 0.05 | 214 | 0.10 | 227 | 0.10 | 184 | 0.06 | 202 | 0.11 |
| **NC_009334.1** | 172764 | 8 | 0.15 | 66 | 2.77 | 13 | 0.05 | 591 | 21.13 | 13 | 0.57 | 5 | 0.03 | 27 | 1.26 | 28 | 1.30 | 21 | 0.64 |
| **NC_014075.1** | 3759 | 6 | 0.69 | 22 | 26.20 | 0 | 0.00 | 1 | 0.53 | 1 | 0.51 | 1 | 0.51 | 10 | 0.59 | 0 | 0.00 | 2 | 0.51 |
| **NC_014078.1** | 3808 | 3 | 2.68 | 21 | 25.34 | 10 | 4.49 | 0 | 0.00 | 2 | 1.94 | 0 | 0.00 | 0 | 0.00 | 0 | 0.00 | 0 | 0.00 |
| **NC_002076.2** | 3852 | 1 | 2.62 | 36 | 24.58 | 4 | 4.54 | 12 | 10.49 | 2 | 3.82 | 0 | 0.00 | 0 | 0.00 | 0 | 0.00 | 0 | 0.00 |
| **NC_043415.1** | 3847 | 9 | 6.65 | 60 | 22.46 | 13 | 7.62 | 6 | 6.37 | 2 | 3.77 | 0 | 0.00 | 3 | 4.45 | 2 | 4.81 | 0 | 0.00 |
| **NC_038343.1** | 3246 | 30 | 45.66 | 18 | 22.00 | 0 | 0.00 | 2 | 2.77 | 4 | 6.69 | 0 | 0.00 | 0 | 0.00 | 8 | 14.39 | 0 | 0.00 |
| **NC_038338.1** | 3193 | 5 | 3.16 | 17 | 16.94 | 0 | 0.00 | 2 | 3.91 | 0 | 0.00 | 6 | 9.71 | 0 | 0.00 | 4 | 4.79 | 1 | 3.16 |
| **NC_014076.1** | 3770 | 0 | 0.00 | 6 | 9.47 | 0 | 0.00 | 0 | 0.00 | 2 | 1.33 | 1 | 1.62 | 0 | 0.00 | 0 | 0.00 | 0 | 0.00 |
| **NC_014073.1** | 3629 | 0 | 0.00 | 5 | 8.05 | 0 | 0.00 | 4 | 5.54 | 0 | 0.00 | 0 | 0.00 | 0 | 0.00 | 0 | 0.00 | 0 | 0.00 |
| **NC_038344.1** | 3676 | 0 | 0.00 | 6 | 7.48 | 0 | 0.00 | 35 | 30.20 | 6 | 11.34 | 0 | 0.00 | 0 | 0.00 | 2 | 3.75 | 0 | 0.00 |
| **NC_014084.1** | 3790 | 16 | 6.15 | 18 | 5.96 | 4 | 3.75 | 19 | 8.39 | 4 | 5.33 | 3 | 4.01 | 0 | 0.00 | 11 | 5.80 | 2 | 2.66 |
| **NC_015783.1** | 3725 | 0 | 0.00 | 3 | 5.42 | 0 | 0.00 | 17 | 10.09 | 7 | 5.05 | 0 | 0.00 | 0 | 0.00 | 0 | 0.00 | 0 | 0.00 |
| **NC_014096.1** | 3787 | 5 | 3.72 | 6 | 5.33 | 0 | 0.00 | 2 | 3.59 | 2 | 4.17 | 0 | 0.00 | 0 | 0.00 | 0 | 0.00 | 0 | 0.00 |
| **NC_038337.1** | 3064 | 0 | 0.00 | 3 | 1.40 | 0 | 0.00 | 0 | 0.00 | 0 | 0.00 | 0 | 0.00 | 0 | 0.00 | 0 | 0.00 | 0 | 0.00 |
| **NC_038342.1** | 3301 | 0 | 0.00 | 1 | 0.91 | 4 | 3.06 | 0 | 0.00 | 2 | 2.57 | 0 | 0.00 | 0 | 0.00 | 1 | 1.00 | 0 | 0.00 |
| **NC_015049.1** | 124335 | 1 | 0.02 | 1 | 0.02 | 0 | 0.00 | 10 | 0.08 | 5 | 0.03 | 0 | 0.00 | 0 | 0.00 | 1 | 0.02 | 1 | 0.02 |
| **NC_043413.1** | 3249 | 0 | 0.00 | 0 | 0.00 | 0 | 0.00 | 1 | 2.25 | 0 | 0.00 | 0 | 0.00 | 0 | 0.00 | 1 | 0.58 | 0 | 0.00 |
| **NC_007822.1** | 6068 | 0 | 0.00 | 0 | 0.00 | 0 | 0.00 | 0 | 0.00 | 0 | 0.00 | 0 | 0.00 | 5 | 0.36 | 0 | 0.00 | 0 | 0.00 |
| **NC_007817.1** | 5486 | 0 | 0.00 | 0 | 0.00 | 0 | 0.00 | 2 | 0.35 | 0 | 0.00 | 0 | 0.00 | 0 | 0.00 | 0 | 0.00 | 0 | 0.00 |
| **NC_026662.1** | 3907 | 0 | 0.00 | 0 | 0.00 | 0 | 0.00 | 2 | 0.49 | 0 | 0.00 | 0 | 0.00 | 0 | 0.00 | 0 | 0.00 | 0 | 0.00 |
| **NC_026663.1** | 3612 | 0 | 0.00 | 0 | 0.00 | 0 | 0.00 | 0 | 0.00 | 4 | 0.53 | 0 | 0.00 | 0 | 0.00 | 0 | 0.00 | 0 | 0.00 |
| **NC_038860.1** | 2580 | 0 | 0.00 | 0 | 0.00 | 0 | 0.00 | 1 | 0.74 | 0 | 0.00 | 0 | 0.00 | 0 | 0.00 | 0 | 0.00 | 0 | 0.00 |

**Supplementary Table 6.** Count of non-duplicated reads mapping to each reference sequence and coverage of base pairs (%) of PVF patients.

|  |  | **FVP** | | | | | | | | | | | | | | | | | | | | | |
| --- | --- | --- | --- | --- | --- | --- | --- | --- | --- | --- | --- | --- | --- | --- | --- | --- | --- | --- | --- | --- | --- | --- | --- |
|  |  | sample_118z | | sample_119l | | sample_120s | | sample_121i | | sample_121j | | sample_123u | | sample_124c | | sample_125a | | sample_125k | | sample_125x | | sample_126b | |
| **Genbank ID** | **Length** | **reads** | **cov (%)** | **reads** | **cov (%)** | **reads** | **cov (%)** | **reads** | **cov (%)** | **reads** | **cov (%)** | **reads** | **cov (%)** | **reads** | **cov (%)** | **reads** | **cov (%)** | **reads** | **cov (%)** | **reads** | **cov (%)** | **reads** | **cov (%)** |
| **NC_022518.1** | 9472 | 19887 | 77.45 | 14452 | 75.44 | 18344 | 72.33 | 15988 | 79.30 | 14444 | 73.93 | 16780 | 77.02 | 16226 | 79.19 | 16552 | 76.34 | 19172 | 76.13 | 18863 | 83.79 | 18950 | 79.70 |
| **NC_001422.1** | 5386 | 29 | 36.63 | 15 | 21.44 | 30 | 36.22 | 24 | 29.86 | 22 | 25.05 | 28 | 34.63 | 13 | 13.93 | 55 | 56.55 | 20 | 26.16 | 47 | 51.32 | 42 | 47.96 |
| **NC_038336.1** | 3229 | 10 | 7.53 | 0 | 0.00 | 0 | 0.00 | 0 | 0.00 | 4 | 7.93 | 0 | 0.00 | 0 | 0.00 | 7 | 4.86 | 0 | 0.00 | 0 | 0.00 | 0 | 0.00 |
| **NC_014080.1** | 3736 | 11 | 5.46 | 0 | 0.00 | 0 | 0.00 | 0 | 0.00 | 8 | 0.67 | 0 | 0.00 | 0 | 0.00 | 7 | 6.05 | 0 | 0.00 | 0 | 0.00 | 0 | 0.00 |
| **NC_014081.1** | 3748 | 4 | 4.96 | 0 | 0.00 | 0 | 0.00 | 0 | 0.00 | 0 | 0.00 | 0 | 0.00 | 0 | 0.00 | 24 | 33.75 | 0 | 0.00 | 0 | 0.00 | 1 | 2.27 |
| **NC_038340.1** | 3234 | 3 | 4.61 | 0 | 0.00 | 0 | 0.00 | 0 | 0.00 | 2 | 3.12 | 0 | 0.00 | 1 | 3.12 | 12 | 17.75 | 0 | 0.00 | 0 | 0.00 | 0 | 0.00 |
| **NC_038341.1** | 3153 | 7 | 4.60 | 0 | 0.00 | 0 | 0.00 | 0 | 0.00 | 4 | 8.56 | 0 | 0.00 | 0 | 0.00 | 7 | 5.23 | 0 | 0.00 | 0 | 0.00 | 2 | 2.57 |
| **NC_014091.1** | 3818 | 19 | 4.03 | 0 | 0.00 | 0 | 0.00 | 0 | 0.00 | 6 | 7.28 | 5 | 5.29 | 20 | 27.03 | 27 | 28.84 | 16 | 22.76 | 1 | 2.65 | 3 | 3.64 |
| **NC_038339.1** | 3312 | 1 | 3.05 | 0 | 0.00 | 0 | 0.00 | 0 | 0.00 | 7 | 7.31 | 0 | 0.00 | 3 | 7.97 | 12 | 14.31 | 0 | 0.00 | 0 | 0.00 | 0 | 0.00 |
| **NC_014069.1** | 3690 | 2 | 1.73 | 0 | 0.00 | 0 | 0.00 | 0 | 0.00 | 0 | 0.00 | 0 | 0.00 | 0 | 0.00 | 0 | 0.00 | 0 | 0.00 | 0 | 0.00 | 0 | 0.00 |
| **NC_001716.2** | 153080 | 529 | 1.66 | 719 | 1.82 | 730 | 1.58 | 636 | 1.56 | 433 | 1.59 | 922 | 2.37 | 764 | 1.64 | 777 | 1.53 | 853 | 1.68 | 897 | 1.62 | 874 | 2.06 |
| **NC_014079.1** | 3798 | 13 | 1.29 | 0 | 0.00 | 0 | 0.00 | 0 | 0.00 | 1 | 0.50 | 0 | 0.00 | 0 | 0.00 | 0 | 0.00 | 2 | 0.50 | 0 | 0.00 | 0 | 0.00 |
| **NC_014094.1** | 3705 | 2 | 0.65 | 0 | 0.00 | 0 | 0.00 | 0 | 0.00 | 0 | 0.00 | 0 | 0.00 | 0 | 0.00 | 3 | 1.32 | 0 | 0.00 | 0 | 0.00 | 0 | 0.00 |
| **NC_043414.1** | 3313 | 4 | 0.57 | 0 | 0.00 | 0 | 0.00 | 0 | 0.00 | 7 | 7.24 | 0 | 0.00 | 10 | 13.52 | 45 | 35.19 | 0 | 0.00 | 0 | 0.00 | 5 | 4.86 |
| **NC_026764.1** | 3774 | 2 | 0.56 | 0 | 0.00 | 0 | 0.00 | 0 | 0.00 | 0 | 0.00 | 4 | 1.01 | 9 | 1.17 | 1 | 0.53 | 0 | 0.00 | 0 | 0.00 | 0 | 0.00 |
| **NC_026765.1** | 3621 | 4 | 0.55 | 2 | 0.52 | 14 | 0.61 | 16 | 0.58 | 16 | 0.58 | 2 | 0.52 | 6 | 0.55 | 9 | 0.61 | 17 | 0.58 | 1 | 0.52 | 4 | 0.55 |
| **NC_014074.1** | 3729 | 1 | 0.54 | 0 | 0.00 | 2 | 0.56 | 0 | 0.00 | 0 | 0.00 | 2 | 0.56 | 0 | 0.00 | 5 | 4.64 | 0 | 0.00 | 5 | 0.56 | 1 | 0.51 |
| **NC_043058.1** | 21614 | 381 | 0.40 | 466 | 0.31 | 509 | 0.41 | 294 | 0.40 | 267 | 0.32 | 434 | 0.32 | 342 | 0.31 | 447 | 0.31 | 427 | 0.32 | 362 | 0.31 | 435 | 0.33 |
| **NC_032111.1** | 163005 | 586 | 0.24 | 531 | 0.26 | 791 | 0.27 | 536 | 0.21 | 673 | 0.24 | 702 | 0.26 | 614 | 0.23 | 580 | 0.23 | 670 | 0.25 | 536 | 0.25 | 594 | 0.27 |
| **NC_009333.1** | 137969 | 25 | 0.20 | 3 | 0.03 | 22 | 0.20 | 13 | 0.16 | 18 | 0.18 | 7 | 0.09 | 10 | 0.09 | 4 | 0.06 | 6 | 0.04 | 18 | 0.17 | 12 | 0.17 |
| **NC_008168.1** | 104710 | 9 | 0.15 | 16 | 0.17 | 22 | 0.21 | 11 | 0.13 | 19 | 0.21 | 16 | 0.15 | 4 | 0.08 | 10 | 0.13 | 13 | 0.18 | 22 | 0.17 | 19 | 0.21 |
| **NC_007605.1** | 171823 | 82 | 0.08 | 73 | 0.14 | 75 | 0.43 | 174 | 0.20 | 84 | 1.35 | 85 | 0.11 | 94 | 0.10 | 66 | 0.33 | 95 | 0.29 | 75 | 0.13 | 222 | 0.14 |
| **NC_006146.1** | 171096 | 212 | 0.07 | 225 | 0.05 | 245 | 0.09 | 168 | 0.08 | 188 | 0.06 | 206 | 0.06 | 199 | 0.09 | 232 | 0.08 | 193 | 0.07 | 173 | 0.06 | 198 | 0.06 |
| **NC_009334.1** | 172764 | 8 | 0.04 | 14 | 0.06 | 6 | 0.02 | 6 | 0.04 | 52 | 2.13 | 17 | 0.06 | 10 | 0.13 | 8 | 0.42 | 4 | 0.12 | 4 | 0.06 | 7 | 0.02 |
| **NC_014075.1** | 3759 | 0 | 0.00 | 2 | 0.51 | 1 | 0.51 | 0 | 0.00 | 7 | 10.08 | 0 | 0.00 | 0 | 0.00 | 25 | 34.88 | 0 | 0.00 | 0 | 0.00 | 0 | 0.00 |
| **NC_014078.1** | 3808 | 0 | 0.00 | 1 | 0.53 | 0 | 0.00 | 0 | 0.00 | 0 | 0.00 | 0 | 0.00 | 0 | 0.00 | 30 | 42.28 | 0 | 0.00 | 0 | 0.00 | 0 | 0.00 |
| **NC_002076.2** | 3852 | 0 | 0.00 | 0 | 0.00 | 0 | 0.00 | 0 | 0.00 | 0 | 0.00 | 0 | 0.00 | 1 | 0.49 | 16 | 13.16 | 2 | 3.30 | 0 | 0.00 | 0 | 0.00 |
| **NC_043415.1** | 3847 | 0 | 0.00 | 0 | 0.00 | 0 | 0.00 | 0 | 0.00 | 33 | 8.53 | 0 | 0.00 | 39 | 28.67 | 18 | 8.71 | 0 | 0.00 | 1 | 2.63 | 9 | 2.89 |
| **NC_038343.1** | 3246 | 0 | 0.00 | 0 | 0.00 | 0 | 0.00 | 0 | 0.00 | 4 | 11.86 | 0 | 0.00 | 0 | 0.00 | 6 | 10.94 | 0 | 0.00 | 0 | 0.00 | 0 | 0.00 |
| **NC_038338.1** | 3193 | 0 | 0.00 | 0 | 0.00 | 0 | 0.00 | 0 | 0.00 | 6 | 1.19 | 0 | 0.00 | 1 | 3.16 | 18 | 16.60 | 0 | 0.00 | 0 | 0.00 | 0 | 0.00 |
| **NC_014076.1** | 3770 | 0 | 0.00 | 0 | 0.00 | 0 | 0.00 | 0 | 0.00 | 6 | 6.84 | 0 | 0.00 | 1 | 1.99 | 20 | 23.50 | 0 | 0.00 | 0 | 0.00 | 0 | 0.00 |
| **NC_014073.1** | 3629 | 0 | 0.00 | 0 | 0.00 | 0 | 0.00 | 0 | 0.00 | 0 | 0.00 | 0 | 0.00 | 0 | 0.00 | 5 | 8.90 | 0 | 0.00 | 0 | 0.00 | 0 | 0.00 |
| **NC_038344.1** | 3676 | 0 | 0.00 | 0 | 0.00 | 0 | 0.00 | 0 | 0.00 | 3 | 3.29 | 0 | 0.00 | 0 | 0.00 | 24 | 21.35 | 0 | 0.00 | 0 | 0.00 | 0 | 0.00 |
| **NC_014084.1** | 3790 | 0 | 0.00 | 0 | 0.00 | 0 | 0.00 | 0 | 0.00 | 4 | 3.03 | 0 | 0.00 | 18 | 5.01 | 27 | 7.41 | 0 | 0.00 | 0 | 0.00 | 4 | 5.22 |
| **NC_015783.1** | 3725 | 0 | 0.00 | 0 | 0.00 | 0 | 0.00 | 0 | 0.00 | 3 | 5.42 | 0 | 0.00 | 0 | 0.00 | 23 | 24.51 | 0 | 0.00 | 0 | 0.00 | 0 | 0.00 |
| **NC_014096.1** | 3787 | 0 | 0.00 | 0 | 0.00 | 0 | 0.00 | 0 | 0.00 | 17 | 18.19 | 0 | 0.00 | 17 | 17.69 | 8 | 7.16 | 0 | 0.00 | 0 | 0.00 | 24 | 17.88 |
| **NC_038337.1** | 3064 | 0 | 0.00 | 0 | 0.00 | 3 | 0.65 | 0 | 0.00 | 2 | 2.58 | 0 | 0.00 | 0 | 0.00 | 12 | 3.62 | 0 | 0.00 | 0 | 0.00 | 0 | 0.00 |
| **NC_038342.1** | 3301 | 0 | 0.00 | 0 | 0.00 | 0 | 0.00 | 0 | 0.00 | 0 | 0.00 | 0 | 0.00 | 0 | 0.00 | 0 | 0.00 | 0 | 0.00 | 0 | 0.00 | 0 | 0.00 |
| **NC_015049.1** | 124335 | 0 | 0.00 | 4 | 0.03 | 2 | 0.02 | 0 | 0.00 | 1 | 0.02 | 3 | 0.03 | 8 | 0.06 | 1 | 0.02 | 5 | 0.02 | 0 | 0.00 | 1 | 0.02 |
| **NC_043413.1** | 3249 | 0 | 0.00 | 0 | 0.00 | 0 | 0.00 | 0 | 0.00 | 0 | 0.00 | 0 | 0.00 | 0 | 0.00 | 3 | 6.37 | 0 | 0.00 | 1 | 0.58 | 1 | 0.58 |
| **NC_007822.1** | 6068 | 0 | 0.00 | 0 | 0.00 | 2 | 0.31 | 0 | 0.00 | 1 | 0.31 | 1 | 0.31 | 0 | 0.00 | 0 | 0.00 | 0 | 0.00 | 0 | 0.00 | 2 | 0.31 |
| **NC_007817.1** | 5486 | 0 | 0.00 | 0 | 0.00 | 4 | 0.53 | 0 | 0.00 | 0 | 0.00 | 0 | 0.00 | 0 | 0.00 | 0 | 0.00 | 0 | 0.00 | 0 | 0.00 | 0 | 0.00 |
| **NC_026662.1** | 3907 | 0 | 0.00 | 0 | 0.00 | 0 | 0.00 | 0 | 0.00 | 0 | 0.00 | 0 | 0.00 | 0 | 0.00 | 0 | 0.00 | 0 | 0.00 | 0 | 0.00 | 0 | 0.00 |
| **NC_026663.1** | 3612 | 0 | 0.00 | 0 | 0.00 | 0 | 0.00 | 0 | 0.00 | 0 | 0.00 | 0 | 0.00 | 0 | 0.00 | 0 | 0.00 | 0 | 0.00 | 0 | 0.00 | 0 | 0.00 |
| **NC_038860.1** | 2580 | 0 | 0.00 | 0 | 0.00 | 0 | 0.00 | 0 | 0.00 | 0 | 0.00 | 0 | 0.00 | 0 | 0.00 | 0 | 0.00 | 0 | 0.00 | 0 | 0.00 | 0 | 0.00 |

**Supplementary Table 7.** Top 10 most frequent relative entries.

| **Phylum** | **Class** | **Order** | **Family** | **Genus** | **Species** | **GenBank ID** | **non-FVP** | **PVF** |
| --- | --- | --- | --- | --- | --- | --- | --- | --- |
| Artverviricota | Revtraviricetes | Ortervirales | Retroviridae | Human endogenous retroviruses | Human endogenous retrovirus K | NC_022518.1 | 0.8692 | 0.8856 |
| Peploviricota | Herviviricetes | Herpesvirales | Herpesviridae | Roseolovirus | Human betaherpesvirus 7 | NC_001716.2 | 0.0438 | 0.0380 |
| Nucleocytoviricota | Pokkesviricetes | Chitovirales | Poxviridae | Chordopoxvirinae_unclassified | BeAn 58058 virus | NC_032111.1 | 0.0348 | 0.0321 |
| Peploviricota | Herviviricetes | Herpesvirales | Herpesviridae | Lymphocryptovirus | Papiine gammaherpesvirus 1 | NC_043058.1 | 0.0197 | 0.0204 |
| Peploviricota | Herviviricetes | Herpesvirales | Herpesviridae | Lymphocryptovirus | Macacine gammaherpesvirus 4 | NC_006146.1 | 0.0091 | 0.0106 |
| Peploviricota | Herviviricetes | Herpesvirales | Herpesviridae | Lymphocryptovirus | Human gammaherpesvirus 4 | NC_007605.1 | 0.0098 | 0.0053 |
| Peploviricota | Herviviricetes | Herpesvirales | Herpesviridae | Lymphocryptovirus | Human gammaherpesvirus 4 | NC_009334.1 | 0.0035 | 0.0007 |
| Phixviricota | Malgrandaviricetes | Petitvirales | Microviridae | Sinsheimervirus | Escherichia virus phiX174 | NC_001422.1 | 0.0022 | 0.0015 |
| Peploviricota | Herviviricetes | Herpesvirales | Herpesviridae | Rhadinovirus | Human gammaherpesvirus 8 | NC_009333.1 | 0.0014 | 0.0006 |
|  |  |  | Baculoviridae | Betabaculovirus | Choristoneura fumiferana granulovirus | NC_008168.1 | 0.0009 | 0.0008 |

**Supplementary Table 8.** Relative frequency table for all GenBank entries.

| **Phylum** | **Class** | **Family** | **Genus** | **Species** | **GenBank ID** | **non-FVP** | **PVF** |
| --- | --- | --- | --- | --- | --- | --- | --- |
| Artverviricota | Revtraviricetes | Retroviridae | Human endogenous retroviruses | Human endogenous retrovirus K | NC_022518.1 | 0.8692 | 0.8856 |
| Peploviricota | Herviviricetes | Herpesviridae | Roseolovirus | Human betaherpesvirus 7 | NC_001716.2 | 0.0438 | 0.0380 |
| Nucleocytoviricota | Pokkesviricetes | Poxviridae | Chordopoxvirinae_unclassified | BeAn 58058 virus | NC_032111.1 | 0.0348 | 0.0321 |
| Peploviricota | Herviviricetes | Herpesviridae | Lymphocryptovirus | Papiine gammaherpesvirus 1 | NC_043058.1 | 0.0197 | 0.0204 |
| Peploviricota | Herviviricetes | Herpesviridae | Lymphocryptovirus | Macacine gammaherpesvirus 4 | NC_006146.1 | 0.0091 | 0.0106 |
| Peploviricota | Herviviricetes | Herpesviridae | Lymphocryptovirus | Human gammaherpesvirus 4 | NC_007605.1 | 0.0098 | 0.0053 |
|  |  | Anelloviridae | Alphatorquevirus | Torque teno virus 24 | NC_038343.1 | 0.0003 | 0.0001 |
| Phixviricota | Malgrandaviricetes | Microviridae | Sinsheimervirus | Escherichia virus phiX174 | NC_001422.1 | 0.0022 | 0.0015 |
|  |  | Anelloviridae | Alphatorquevirus | Torque teno virus 18 | NC_043414.1 | 0.0006 | 0.0003 |
|  |  | Anelloviridae | Alphatorquevirus | Torque teno virus 8 | NC_014084.1 | 0.0004 | 0.0003 |
|  |  | Baculoviridae | Betabaculovirus | Choristoneura fumiferana granulovirus | NC_008168.1 | 0.0009 | 0.0008 |
| Peploviricota | Herviviricetes | Herpesviridae | Rhadinovirus | Human gammaherpesvirus 8 | NC_009333.1 | 0.0014 | 0.0006 |
|  |  | Anelloviridae | Alphatorquevirus | Torque teno virus 13 | NC_038339.1 | 0.0003 | 0.0001 |
|  |  | Anelloviridae | Alphatorquevirus | Torque teno virus 22 | NC_043415.1 | 0.0005 | 0.0005 |
| Peploviricota | Herviviricetes | Herpesviridae | Lymphocryptovirus | Human gammaherpesvirus 4 | NC_009334.1 | 0.0035 | 0.0007 |
|  |  | Anelloviridae | Alphatorquevirus | Torque teno virus 16 | NC_014091.1 | 0.0008 | 0.0004 |
|  |  | Anelloviridae | Alphatorquevirus | Simian torque teno virus 34 | NC_026765.1 | 0.0003 | 0.0004 |
|  |  | Anelloviridae | Alphatorquevirus | Torque teno virus 12 | NC_014075.1 | 0.0002 | 0.0002 |
|  |  | Anelloviridae | Alphatorquevirus | Torque teno virus 15 | NC_014096.1 | 0.0001 | 0.0003 |
|  |  | Anelloviridae | Alphatorquevirus | Torque teno virus 11 | NC_038338.1 | 0.0002 | 0.0001 |
|  |  | Anelloviridae | Alphatorquevirus | Torque teno virus 3 | NC_014081.1 | 0.0001 | 0.0001 |
|  |  | Anelloviridae | Alphatorquevirus | Torque teno virus 27 | NC_014074.1 | 0.0001 | 0.0001 |
|  |  | Anelloviridae | Alphatorquevirus | Torque teno virus 19 | NC_014078.1 | 0.0002 | 0.0001 |
|  |  | Anelloviridae | Alphatorquevirus | Torque teno virus 5 | NC_038336.1 | 0.0001 | 0.0001 |
|  |  | Anelloviridae | Alphatorquevirus | Torque teno virus 21 | NC_038341.1 | 0.0001 | 0.0001 |
|  |  | Anelloviridae | Alphatorquevirus | Torque teno virus 20 | NC_038340.1 | 0.0001 | 0.0001 |
|  |  | Anelloviridae | Alphatorquevirus | Torque teno virus 1 | NC_002076.2 | 0.0003 | 0.0001 |
|  |  | Anelloviridae | Alphatorquevirus | Torque teno virus 26 | NC_014079.1 | 0.0000 | 0.0001 |
|  |  | Anelloviridae | Alphatorquevirus | Torque teno virus 7 | NC_014080.1 | 0.0001 | 0.0001 |
|  |  | Anelloviridae | Alphatorquevirus | Torque teno virus 6 | NC_014094.1 | 0.0000 | 0.0000 |
| Peploviricota | Herviviricetes | Herpesviridae | Rhadinovirus | Cricetid gammaherpesvirus 2 | NC_015049.1 | 0.0001 | 0.0001 |
|  |  | Anelloviridae | Alphatorquevirus | Torque teno virus 10 | NC_014076.1 | 0.0000 | 0.0001 |
| Phixviricota | Malgrandaviricetes | Microviridae | Gequatrovirus | Escherichia virus Talmos Escherichia phageID2  Moscow/ID/2001 | NC_007817.1 | 0.0000 | 0.0000 |
| Phixviricota | Malgrandaviricetes | Microviridae | Alphatrevirus | Escherichia virus WA45 | NC_007822.1 | 0.0000 | 0.0000 |
|  |  | Anelloviridae | Alphatorquevirus | Torque teno virus 4 | NC_014069.1 | 0.0000 | 0.0000 |
|  |  | Anelloviridae | Alphatorquevirus | Torque teno virus 28 | NC_014073.1 | 0.0000 | 0.0000 |
|  |  | Anelloviridae | Alphatorquevirus_unclassified | Torque teno virus | NC_015783.1 | 0.0001 | 0.0001 |
|  |  | Anelloviridae | Alphatorquevirus | Simian Torque teno virus 30 | NC_026663.1 | 0.0000 | 0.0000 |
|  |  | Anelloviridae | Alphatorquevirus | Simian Torque teno virus 33 | NC_026764.1 | 0.0000 | 0.0001 |
|  |  | Anelloviridae | Alphatorquevirus | Torque teno virus 9 | NC_038337.1 | 0.0000 | 0.0001 |
|  |  | Anelloviridae | Alphatorquevirus | Torque teno virus 23 | NC_038342.1 | 0.0000 | 0.0000 |
|  |  | Anelloviridae | Alphatorquevirus | Torque teno virus 29 | NC_038344.1 | 0.0002 | 0.0001 |
|  |  | Anelloviridae | Alphatorquevirus | Torque teno virus 17 | NC_043413.1 | 0.0000 | 0.0000 |

**Supplementary Table 9.** Inflammation-related protein from Olink® Inflammation Panel.

| **Assay** | **Uniprot ID** | **Protein Name** | **Assay** | **Uniprot ID** |  |
| --- | --- | --- | --- | --- | --- |
| **IL8** | **P10145** | Interleukin-8 | **IL-18R1** | **Q13478** | Interleukin-18 receptor 1 |
| **VEGFA** | **P15692** | Vascular endothelial growth factor A | **PD-L1** | **Q9NZQ7** | Programmed cell death 1 ligand 1 |
| **CD8A** | **P01732** | T-cell surface glycoprotein CD8 alpha chain | **Beta-NGF** | **P01138** | Beta-nerve growth factor |
| **MCP-3** | **P80098** | C-C motif chemokine 7 | **CXCL5** | **P42830** | C-X-C motif chemokine 5 |
| **GDNF** | **P39905** | Glial cell line-derived neurotrophic factor | **TRANCE** | **O14788** | Tumor necrosis factor ligand superfamily member 11 |
| **CDCP1** | **Q9H5V8** | CUB domain-containing protein 1 | **HGF** | **P14210** | Hepatocyte growth factor |
| **CD244** | **Q9BZW8** | Natural killer cell receptor 2B4 | **IL-12B** | **P29460** | Interleukin-12 subunit beta |
| **IL7** | **P13232** | Interleukin-7 | **IL-24** | **Q13007** | Interleukin-24 |
| **OPG** | **O00300** | Osteoprotegerin (OPG); Tumor necrosis factor receptor superfamily member 11B | **IL13** | **P35225** | Interleukin-13 |
| **LAP TGF-beta-1** | **P01137** | Transforming growth factor beta-1 proprotein | **ARTN** | **Q5T4W7** | Artemin |
| **uPA** | **P00749** | Urokinase-type plasminogen activator | **MMP-10** | **P09238** | Stromelysin-2 |
| **IL6** | **P05231** | Interleukin-6 | **IL10** | **P22301** | Interleukin-10 |
| **IL-17C** | **Q9P0M4** | Interleukin-17C | **TNF** | **P01375** | Tumor necrosis factor |
| **MCP-1** | **P13500** | C-C motif chemokine 2 | **CCL23** | **P55773** | C-C motif chemokine 23 |
| **IL-17A** | **Q16552** | Interleukin-17A | **CD5** | **P06127** | T-cell surface glycoprotein CD5 |
| **CXCL11** | **O14625** | C-X-C motif chemokine 11 | **CCL3** | **P10147** | C-C motif chemokine 3 |
| **AXIN1** | **O15169** | Axin-1 | **Flt3L** | **P49771** | Fms-related tyrosine kinase 3 ligand |
| **TRAIL** | **P50591** | Tumor necrosis factor ligand superfamily member 10 | **CXCL6** | **P80162** | C-X-C motif chemokine 6 |
| **IL-20RA** | **Q9UHF4** | Interleukin-20 receptor subunit alpha | **CXCL10** | **P02778** | C-X-C motif chemokine 10 |
| **CXCL9** | **Q07325** | C-X-C motif chemokine 9 | **4E-BP1** | **Q13541** | Eukaryotic translation initiation factor 4E-binding protein 1 |
| **CST5** | **P28325** | Cystatin-D | **IL-20** | **Q9NYY1** | Interleukin-20 |
| **IL-2RB** | **P14784** | Interleukin-2 receptor subunit beta | **SIRT2** | **Q8IXJ6** | NAD-dependent protein deacetylase sirtuin-2 |
| **IL-1 alpha** | **P01583** | Interleukin-1 alpha | **CCL28** | **Q9NRJ3** | C-C motif chemokine 28 |
| **OSM** | **P13725** | Oncostatin-M | **DNER** | **Q8NFT8** | Delta and Notch-like epidermal growth factor-related receptor |
| **IL2** | **P60568** | Interleukin-2 | **EN-RAGE** | **P80511** | Protein S100-A12 |
| **CXCL1** | **P09341** | Growth-regulated alpha protein | **CD40** | **P25942** | Tumor necrosis factor receptor superfamily member 5 |
| **TSLP** | **Q969D9** | Thymic stromal lymphopoietin | **IL33** | **O95760** | Interleukin-33 |
| **CCL4** | **P13236** | C-C motif chemokine 4 | **IFN-gamma** | **P01579** | Interferon gamma |
| **CD6** | **P30203** | T-cell differentiation antigen CD6 | **FGF-19** | **O95750** | Fibroblast growth factor 19 |
| **SCF** | **P21583** | Kit ligand | **IL4** | **P05112** | Interleukin-4 |
| **IL18** | **Q14116** | Interleukin-18 | **LIF** | **P15018** | Leukemia inhibitory factor |
| **SLAMF1** | **Q13291** | Signaling lymphocytic activation molecule | **NRTN** | **Q99748** | Neurturin |
| **TGF-alpha** | **P01135** | Protransforming growth factor alpha | **MCP-2** | **P80075** | C-C motif chemokine 8 |
| **MCP-4** | **Q99616** | C-C motif chemokine 13 | **CASP-8** | **Q14790** | Caspase-8 |
| **CCL11** | **P51671** | Eotaxin | **CCL25** | **O15444** | C-C motif chemokine 25 |
| **TNFSF14** | **O43557** | Tumor necrosis factor ligand superfamily member 14 | **CX3CL1** | **P78423** | Fractalkine |
| **FGF-23** | **Q9GZV9** | Fibroblast growth factor 23 | **TNFRSF9** | **Q07011** | Tumor necrosis factor receptor superfamily member 9 |
| **IL-10RA** | **Q13651** | Interleukin-10 receptor subunit alpha | **NT-3** | **P20783** | Neurotrophin-3 |
| **FGF-5** | **P12034** | Fibroblast growth factor 5 | **TWEAK** | **O43508** | Tumor necrosis factor ligand superfamily member 12 |
| **MMP-1** | **P03956** | Interstitial collagenase | **CCL20** | **P78556** | C-C motif chemokine 20 |
| **LIF-R** | **P42702** | Leukemia inhibitory factor receptor | **ST1A1** | **P50225** | Sulfotransferase 1A1 |
| **FGF-21** | **Q9NSA1** | Fibroblast growth factor 21 | **STAMBP** | **O95630** | STAM-binding protein |
| **CCL19** | **Q99731** | C-C motif chemokine 19 | **IL5** | **P05113** | Interleukin-5 |
| **IL-15RA** | **Q13261** | Interleukin-15 receptor subunit alpha | **ADA** | **P00813** | Adenosine deaminase |
| **IL-10RB** | **Q08334** | Interleukin-10 receptor subunit beta | **TNFB** | **P01374** | Lymphotoxin-alpha |
| **IL-22 RA1** | **Q8N6P7** | Interleukin-22 receptor subunit alpha-1 | **CSF-1** | **P09603** | Macrophage colony-stimulating factor 1 |
